# Experience Modulates the Reproductive Response to Heat Stress in *C*. *elegans* via Multiple Physiological Processes

**DOI:** 10.1371/journal.pone.0145925

**Published:** 2015-12-29

**Authors:** Devin Y. Gouvêa, Erin Z. Aprison, Ilya Ruvinsky

**Affiliations:** 1 Committee on Conceptual and Historical Studies of Science, The University of Chicago, Chicago, Illinois, United States of America; 2 Committee on Evolutionary Biology, The University of Chicago, Chicago, Illinois, United States of America; 3 Department of Ecology and Evolution, The University of Chicago, Chicago, Illinois, United States of America; 4 Department of Organismal Biology and Anatomy, The University of Chicago, Chicago, Illinois, United States of America; Centre National de la Recherche Scientifique & University of Nice Sophia-Antipolis, FRANCE

## Abstract

Natural environments are considerably more variable than laboratory settings and often involve transient exposure to stressful conditions. To fully understand how organisms have evolved to respond to any given stress, prior experience must therefore be considered. We investigated the effects of individual and ancestral experience on *C*. *elegans* reproduction. We documented ways in which cultivation at 15°C or 25°C affects developmental time, lifetime fecundity, and reproductive performance after severe heat stress that exceeds the fertile range of the organism but is compatible with survival and future fecundity. We found that experience modulates multiple aspects of reproductive physiology, including the male and female germ lines and the interaction between them. These responses vary in their environmental sensitivity, suggesting the existence of complex mechanisms for coping with unpredictable and stressful environments.

## Introduction

For model organisms, laboratory life is a relatively serene affair—food is generally abundant and the environment stable. Life in the wild seems by contrast to be “one great blooming, buzzing, confusion” [1]. Seasonal and daily rhythms join predators, parasites, food shortages, and severe weather to produce incessant environmental change. But the existence of unpredictable challenges need not be overwhelming to organisms equipped with strategies to handle environmental pressures [2,3]. Depending on the nature of the change [4], developmental, physiological, and behavioral responses range from predictable plasticity [5–7] to stochastic bet-hedging [8–10].

Assessing the relevance of laboratory studies to stress response in the wild is an important but challenging task. Even for well-studied model species like *Caenorhabditis elegans*, basic questions about feeding, migration, and life history remain unanswered [11]. Traits likely to be important for life in natural environments, including responses to dietary stress [12–15], crowding [16], and heat shock [17–19], have been extensively studied in laboratory populations, but their native functions are less well understood. For example, individuals collected in the wild display a number of phenotypes that would be considered pathological in the lab, including constipation, internal bacteria, and abnormal germ cells [20,21]. Local extinctions are frequent [22,23], while non-reproductive dauer larvae [24,25] apparently sustain populations between periods of active expansion and facilitate migration to new food sources [20,21,23]. How then should stress be defined, and its physiological consequences understood?

One way to approach this problem is through systematic laboratory investigation of organismal biology under a broad range of conditions. The goal is to establish the performance limits of physiological systems and the general patterns of their response to stress. Some treatments may not be relevant in natural habitats and some key aspects of native environments will be missing. However, these limitations may be compensated by the benefits of thorough exploration under controlled conditions. We feel such laboratory studies could make useful contributions to a larger research program seeking detailed mechanistic understanding of life in native habitats.

Among the various abiotic stressors affecting nematodes, temperature is probably the most extensively studied in the lab. As growth temperatures increase to 27°C, fertility drops dramatically in the reference laboratory strain N2 and in a variety of wild isolates native to different climates [26–28]. Long-term maintenance becomes impossible at 27°C or 28°C [28,29] and embryogenesis is compromised at temperatures as low as 25°C [28,30,31]. Nevertheless, *C*. *elegans* has been isolated from many locations where ambient temperatures routinely exceed these levels [11,21,22,26,32–35].

How can *C*. *elegans* endure high temperatures in the wild, particularly during periods of population expansion? One possibility is that worms may exploit local temperature heterogeneity. The species has a well-studied thermotaxis response [36–41] and thermal preference has been found to vary substantially among natural isolates [42,43]. Temperatures inside rotting fruits (a major food source) may be either higher or lower than air temperatures, depending on sun exposure [44]. The present study is motivated by another possibility—that worms growing up in rugged natural habitats may be better equipped to handle heat stress than their pampered laboratory cousins. To explore this possibility, we systematically studied the effects of growth temperature on many aspects of the reproductive physiology of *C*. *elegans* and its response to chronic heat stress.

## Results

### Experimental rationale and terminology

We focused on temperatures (28–32°C) at which *C*. *elegans* cannot continuously reproduce [28,29] but can survive prolonged exposure without permanent sterility [27]. To test the effects of prior experience, we raised worms at three temperatures, 15°C, 20°C, and 25°C, commonly used in *C*. *elegans* research. Fecundity has long been known to decline at either end of this range [45,46] but the optimal temperature was unknown until recently. Under standard laboratory conditions, N2 achieves maximum brood sizes slightly above 18°C, with a symmetrical decline in fecundity at both higher and lower temperatures and only a small loss at 20°C [28]. To provide a baseline for further study, we first report the effects of our rearing temperatures on developmental time and lifetime fecundity. We then establish their effects on reproductive performance after 24 hours at temperatures between 28°C and 32°C. Measured phenotypes include the ability of adults to recover fecundity, the ability of embryos to hatch, and gonad health during recovery. We finally describe the effects of ancestral exposure to the severe stress treatment. All raw data are provided in S1–S4 Tables.

Most of our experiments involve shifting individual hermaphrodites between temperatures. Different stages of the *C*. *elegans* life cycle are known to exhibit different levels of sensitivity to environmental stressors such as extreme cold [47,48], population density [49], and starvation [15,50]. To decide when to shift our worms, we considered how best to deconstruct the complex reproductive response to temperature stress into comprehensible lower-level processes. Since *C*. *elegans* reproduces primarily by self-fertilization, we were especially interested in the relative contributions of male and female gametes.

Throughout the early larval stages, proliferation of the primordial germ cells produces a common pool of progenitor cells [51–54]. Midway through the L4 stage, the fate of germ cells entering meiosis irreversibly switches from spermatogenesis to oogenesis [54] and spermatogenesis is complete by the end of this stage [55]. To make a reasonable if imperfect distinction between male and female gametes, animals could be shifted anywhere from mid-L4 to early young adulthood. We chose the cellularization of the first oocyte as our landmark because it can be easily and reproducibly identified, and because it happens late enough to avoid interference from the events surrounding the molt and preceding lethargus period. Since the first embryos appear within hours of the first oocyte [27], this landmark doubles as a convenient boundary between the developmental and reproductive phases of the life cycle.

In what follows, we refer to worms at the onset of oocyte cellularization as “young adults” and describe the periods before and afterwards respectively as “development” and “reproduction.” To describe the conditions of worm maintenance more generally we use the terms “growth,” “rearing,” and “cultivation” interchangeably. Since it can be difficult to tell whether the “eggs” laid during stress are fertilized, let alone viable, we use the term generously and reserve “embryo” for fertilized, developing eggs.

### Temperature-dependent developmental scaling

**Fig 1:**
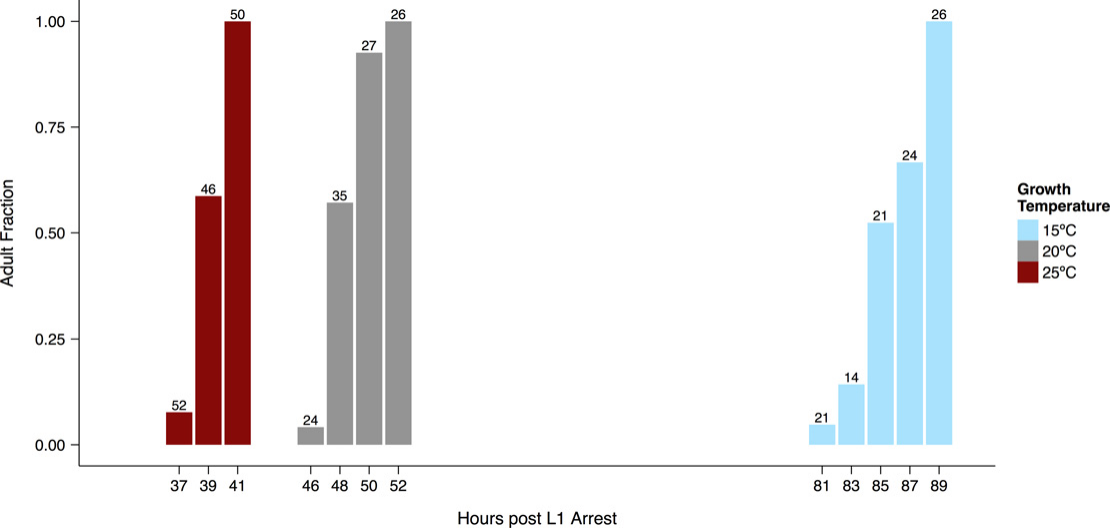
Development time depends on temperature. Bars show the fraction of worms (n indicated above each bar) that had completed the final molt at each observation time (measured from the point at which arrested L1 larvae were placed on food). Animals were not followed continuously, so numbers scored at earlier times may be smaller than those scored at later times. A representative trial is shown for each cultivation temperature. See S1 Fig for additional trials and S1 Table for raw data.

In line with previous reports [45,46], we found that the time required for worms to reach the adult stage was shorter at 20°C than at 15°C, and shorter still at 25°C (Fig 1, S1 Fig). The average time to young adulthood approximately doubled between 25°C and 15°C. At all three temperatures the time required for the entire population to transition from the L4 to the adult stage was on the order of 10% of average time to adulthood. This result is consistent with a straightforward scaling of developmental rate and temperature.

In the same assay, we recorded the number of oocytes in the gonad and the number of fertilized embryos in the uterus, and identified the time at which approximately 50% of individuals had formed their first oocyte. As expected, it took longer to begin oocyte production at colder temperatures, but once it started, this process proceeded rapidly at all temperatures–within roughly two hours populations progressed from most worms having no oocytes to having two or more oocytes (S2 Fig).

There is an important practical lesson to draw from the rapid increase in oocyte cellularization, the variations in timing that we observed between trials (S1 and S2 Figs), and the subtle differences between our results and the classic data on developmental timing [45]. When staging worms for experiments, it is important not to rely solely on absolute time. Instead, we selected individual young adults for experiments when the population had nearly but not entirely completed the final molt.

### Thermal effects on lifetime fecundity

We next sought to establish the effects of growth conditions on the male and female components of the reproductive system. The causes of temperature-related changes in *Caenorhabditis* fecundity are not fully understood and appear to be complex. Whereas N2 hermaphrodites raised at 20°C apparently utilize their sperm with near-perfect efficiency [55], recent studies in both *C*. *elegans* and *C*. *briggsae* have found that sperm count exceeds total hermaphrodite fecundity near the high and low ends of the temperature range at which each species is fertile [56,57].

**Fig 2:**
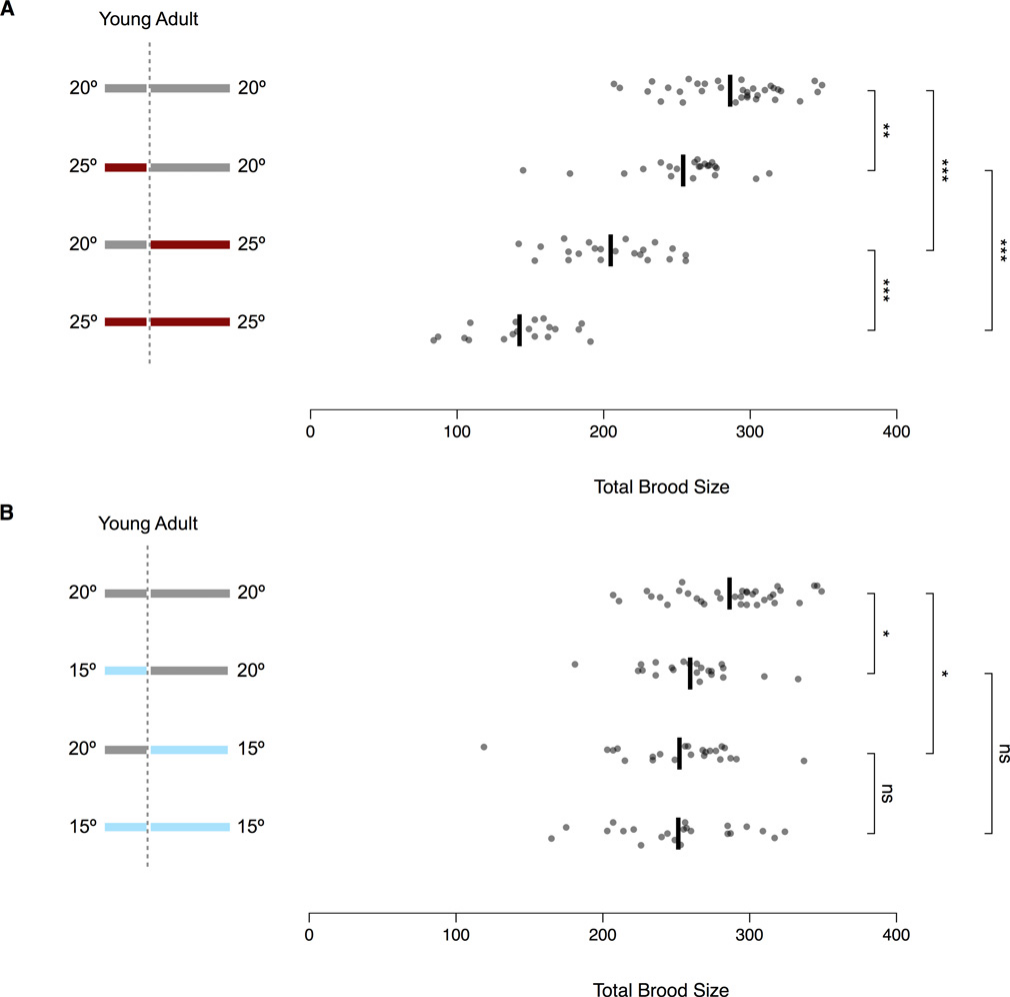
Temperature affects brood size through multiple components of the reproductive system. Lifetime fecundity for temperature treatments, illustrated in the schematics at left, involving (A) 20°C and 25°C or (B) 15°C and 20°C. All shifts were performed just after the onset of oocyte cellularization. Dots represent individual worms (19 ≤ n ≤ 34) and black lines show the mean value for each treatment. The top rows of (A) and (B) show the same data. To avoid the assumption of equal variance across treatments, significance levels were calculated using Welch’s *t*-test, with *p-*values adjusted for multiple testing using the Bonferroni-Holm correction (*p < 0.05, **p < 0.01, *** *p* < 0.001, ns: *p* > 0.05). See S2 Table for raw data.

Measuring the fecundity of worms shifted between temperatures can clarify how thermal stress affects different aspects of reproductive development [26,57,58]. In our implementation of this experimental design, we quantified total lifetime fecundity at 15°C, 20°C, and 25°C and also shifted worms from 20°C to the other two temperatures, and vice versa. For all experiments, hermaphrodites were grown in small populations (see Materials and Methods) from L1 arrest and singled just prior to the start of reproduction, as defined by the onset of oocyte cellularization. For worms kept at the same temperature throughout their lives, total fecundity was highest at 20°C (Fig 2). The loss of fecundity was more severe at 25°C than at 15°C, as would be expected if the optimal temperature is near 18°C [28].

Temperature-shift experiments revealed that thermal stress affects fecundity via processes occurring during both development and reproduction. Worms kept at 25°C for either period had significantly fewer offspring than those kept continually at 20°C, but significantly more than those kept continually at 25°C (Fig 2A). Exposure during reproduction alone was more detrimental than during development alone, and the effects appear to compound with lifelong exposure, possibly in an additive fashion. These results suggest that development at 25°C reduces the number of fertile sperm by lowering either sperm count or sperm fertility, while reproduction at 25°C reduces the production of viable embryos by killing mature sperm, modulating oocyte production, or impairing the interaction between sperm and oocytes.

Worms kept at 15°C during either development or reproduction also had significantly fewer offspring than those kept continually at 20°C (Fig 2B), suggesting that colder temperatures too can affect both the number of fertile sperm and the production of viable embryos. However, the effects of exposure to 15°C during each period were milder than exposure to 25°C and did not appear to compound with lifelong exposure.

### Reproductive effects of severe heat stress

**Fig 3:**
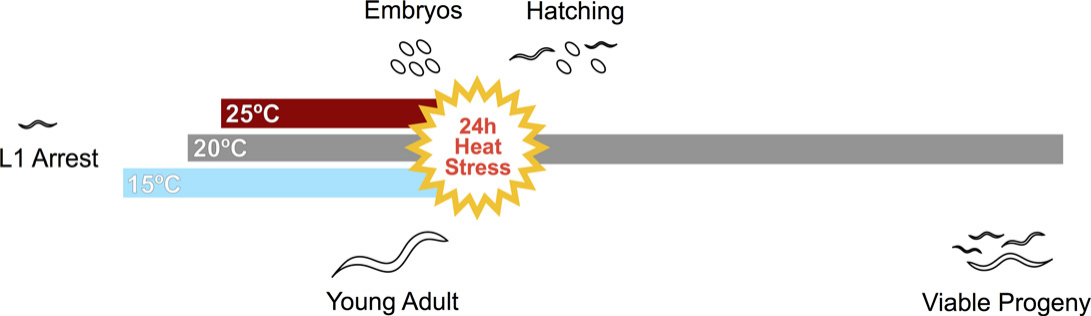
Experimental paradigm for severe heat stress. Worms were transferred to 15°C, 20°C, or 25°C immediately upon plating as arrested L1 larvae (left-hand colored bars). Embryos were collected at the cultivation temperature from adults at the peak of laying (above the bars). Young adults were singled just after the onset of oocyte cellularization (below the bars). We then exposed both embryos and young adults to 24 hours of severe heat stress at temperatures between 28°C and 32°C (yellow circle) and allowed them to recover at 20°C for several days (right-hand gray bar).

We next explored the effects of cultivation temperature on the reproductive response to stresses between 28°C and 32°C, a range in which populations cannot continually reproduce [28,29]. Individual hermaphrodites entering a severe but temporary stress might secure future offspring either by maintaining their own fecundity or by leaving viable embryos. We therefore tested the response of both young adults and freshly laid embryos to a 24-hour heat stress (Fig 3).

In one set of experiments (Fig 3, top), reproducing adults laid embryos on fresh plates for one hour at their cultivation temperature. These plates were then shifted to a stress temperature to assess the ability of embryos to hatch. In another set of experiments (Fig 3, bottom), young adults were singled to fresh plates and shifted to a stress temperature just as they were beginning to produce oocytes. For all experiments, worms were returned to 20°C to recover after 24 hours at the stress temperature.

For both recovery phenotypes, worms raised at 15°C and 25°C exhibited the same general pattern as worms raised at 20°C across a range of stress temperatures [27] (S3 Fig). Despite broad similarities, however, the recovery curves were not identical. Differences among cultivation temperatures were especially pronounced at 29°C and 31°C. We therefore decided to investigate these two stress temperatures more carefully. We also wanted to make sure that the performance of the laboratory adapted N2 strain was representative of the species in general. To do so, we examined reproduction after heat stress in young adults from several strains that were more recently isolated from nature. Despite some strain-specific differences, all but one of the isolates exhibited response curves that were qualitatively similar to N2 (S4 Fig), including a peak in recovery at 31°C [27]. We concluded that N2 represents a reasonable proxy for investigating the effects of temperature on reproductive performance.

### Effects of maternal experience on recovery from severe heat stress

**Fig 4:**
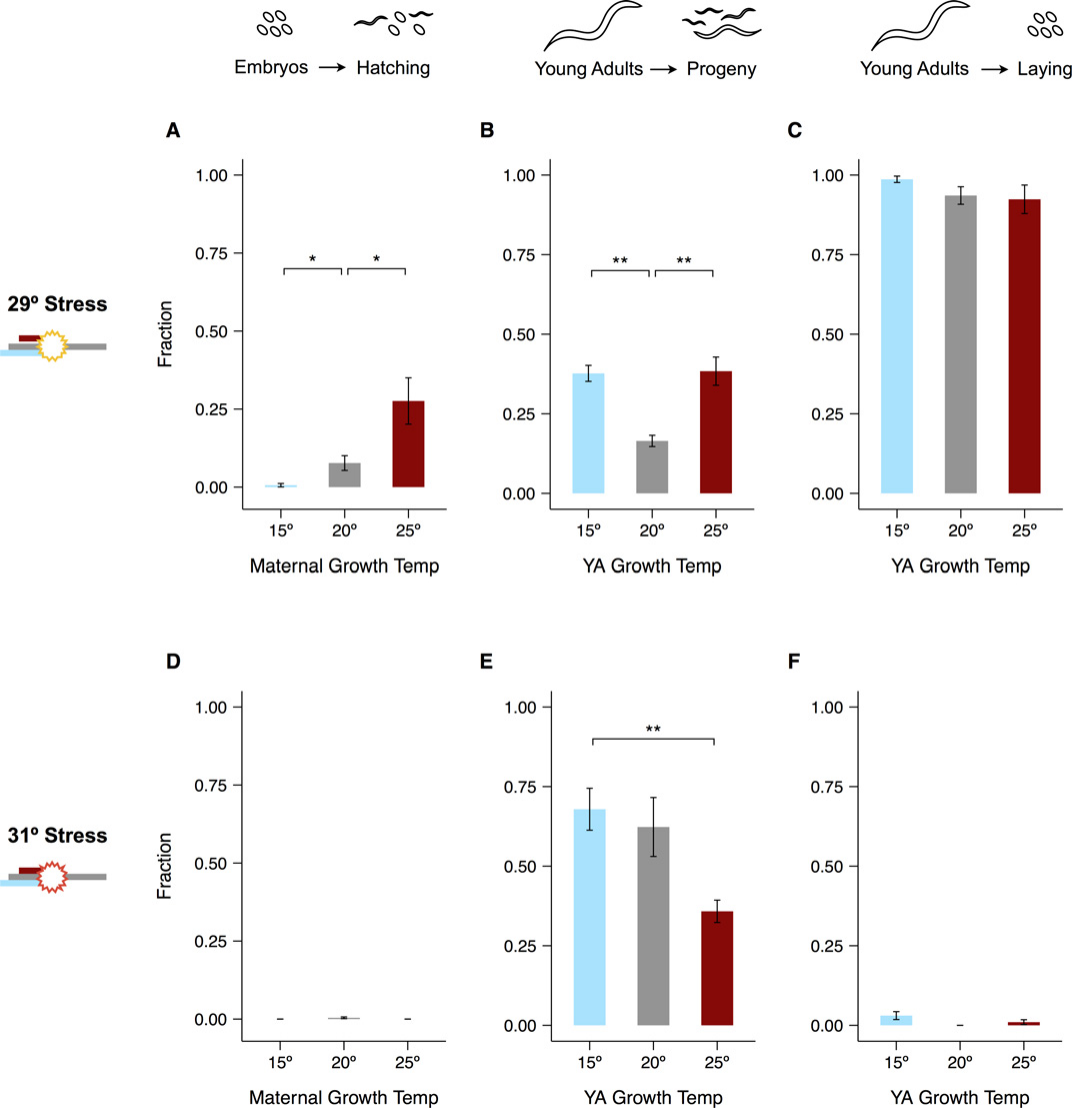
Reproductive effects of severe heat stress. 24-hour stress experiments were conducted at 29°C (A–C) or 31°C (D–F). Hatching of eggs laid by mothers cultivated at different temperatures is shown in (A) and (D). Fractions of adults that recovered live progeny within 5 days after the end of exposure are shown in (B) and (E). Fractions of those same adults that laid eggs during exposure are shown in (C) and (F). Significance levels were calculated using the Mann-Whitney *U*-test with the Bonferroni-Holm correction for multiple testing (*p < 0.05, **p < 0.01). Error bars represent ±1 SEM. See S4 Fig for individual trials and S3 Table for raw data.

The temperature at which worms grew from L1 arrest to young adulthood had considerable but complex effects on their reproductive response to severe heat stress (Fig 4, S5 Fig). We will separately consider the two phenotypes discussed above. The first, hatching of embryos during stress, was strongly dependent on maternal experience at 29°C, with warmer growth temperatures increasing hatching success. By contrast, 31°C exposure appears to have been too harsh to overcome regardless of maternal growth temperature (Fig 4A vs. 4D).

The second phenotype, the ability of young adults to recover fecundity after stress, exemplifies the complex effects of temperature on the reproductive system. After 29°C stress, worms raised at both 15°C and 25°C fared better than those raised at 20°C (Fig 4B). In contrast, worms raised at lower temperatures recovered considerably better after 31°C stress than their counterparts reared at 25°C (Fig 4E). Curiously, worms raised at a given temperature recovered fecundity as frequently or even more frequently after stress at 31°C than after stress at 29°C (Fig 4B vs. 4E). We previously established that 31°C heat stress suppresses ovulation in worms raised at 20°C, which benefits both the male and female components of the reproductive system [27]. Consistent with this finding, nearly all worms in these experiments ceased laying at 31°C regardless of their previous experience, whereas exactly the opposite was true at 29°C (Fig 4C vs. 4F). In contrast to embryos laid at cultivation temperatures and shifted to 29°C, eggs laid during stress almost never hatched, suggesting that fertilization or very early embryogenesis is especially temperature-sensitive.

We interpret these results to mean that the reproductive response to severe heat stress depends on multiple physiological processes that vary in their sensitivity to prior experience. A single generation of cultivation at 15°C or 25°C appears insufficient to affect the viability of eggs fertilized at 29°C, the hatching of embryos shifted to 31°C from cultivation temperatures, or the initiation of ovulation at 31°C. In contrast, the processes determining hatching of embryos shifted to 29°C and the recovery of fecundity at 29°C and 31°C appear to be more sensitive to prior experience. These flexible phenotypes represent attractive targets for understanding how worms have evolved to cope with variable and unpredictable environments.

### Severe heat stress causes extensive sperm damage

To make sense of the complex effects of cultivation temperature on the recovery of fecundity after heat stress, we first focused on the role of sperm. In our experiments, hermaphrodites entered heat stress when spermatogenesis was complete, so spermatids had to survive stress if the worms were to remain fertile. Previous studies of males and hermaphrodites in *C*. *elegans* [26,58] and hermaphrodites in *C*. *briggsae* [57] indicate that heat can damage male gametes during and after spermatogenesis, and that 29°C causes extensive damage to the mature sperm of *C*. *elegans* hermaphrodites raised at 20°C [27].

**Fig 5:**
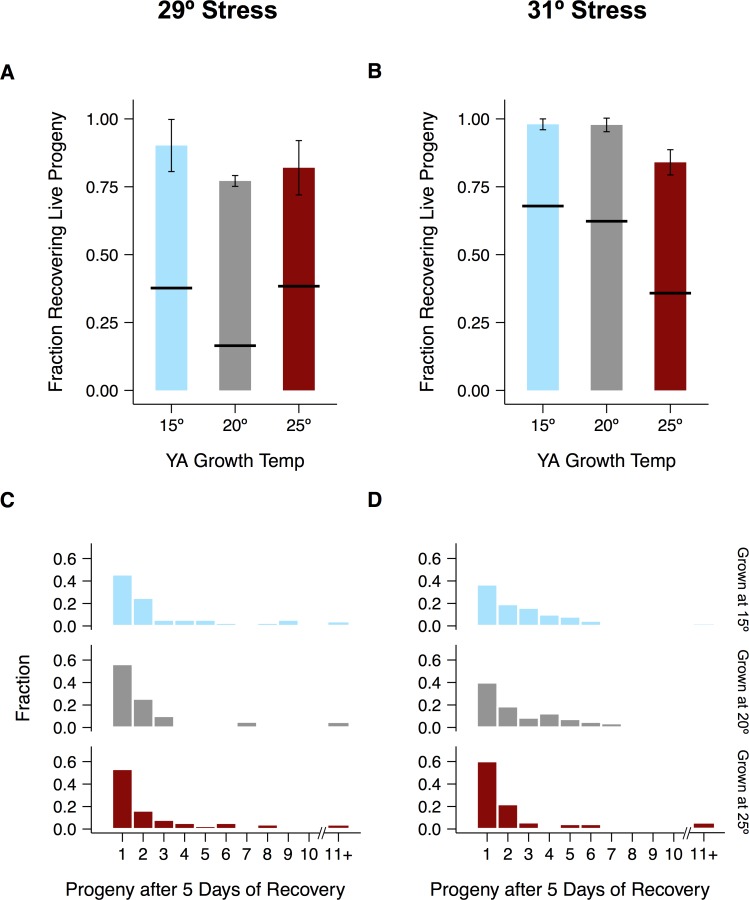
Pervasive sperm damage contributes to low recovery after severe heat stress. 24-hour stress experiments were conducted at 29°C (A, C) or 31°C (B, D). Fractions of pre-reproductive young adults recovering live progeny in the presence of 3 unstressed males are shown in (A) and (B). For comparison, horizontal black lines crossing the bars show the recovery fractions reported in Fig 4B and 4D for hermaphrodites recovering alone. Brood sizes of hermaphrodites recovering alone after stress are shown in (C) and (D). Error bars represent ±1 SEM. See S5 Fig for individual trials and S3 Table for raw data.

Regardless of cultivation temperature, mating with unstressed males dramatically increased the fraction of worms that recovered live progeny (Fig 5A and 5B). This result suggests that most non-recovering hermaphrodites are depleted of functional sperm and would otherwise be able to reproduce. It also helps to explain the small brood sizes observed when hermaphrodites recovered alone (Fig 5C and 5D). By contrast, brood sizes after 29°C stress were an order of magnitude higher following mating with unstressed males (S6 Fig). We concluded that sperm are generally more sensitive to heat stress than the capacity to produce new oocytes, and that a large fraction of them die or lose fertility during heat stress. This sperm damage is a major contributor to the low rate of self-recovery after severe heat stress, and does not appear to be alleviated by cultivation at 15°C or 25°C.

Importantly, even with the benefit of unstressed sperm, not all worms were able to recover fecundity (particularly after the 29°C stress), indicating that other aspects of the reproductive system were irreparably damaged. This conclusion is further supported by the fact that brood sizes of mated worms recovering from 29°C stress did not reach even the low level produced by lifetime cultivation at 25°C (Fig 2A vs. S6 Fig). These results suggest that the complex relationship between cultivation temperature and recovery (Fig 4B and 4D) reflects the interaction of different components of the reproductive system. We next investigated how mild temperature stress might prepare the female components for more severe stress.

### Cultivation temperature modulates gonad damage at 29°C

**Fig 6:**
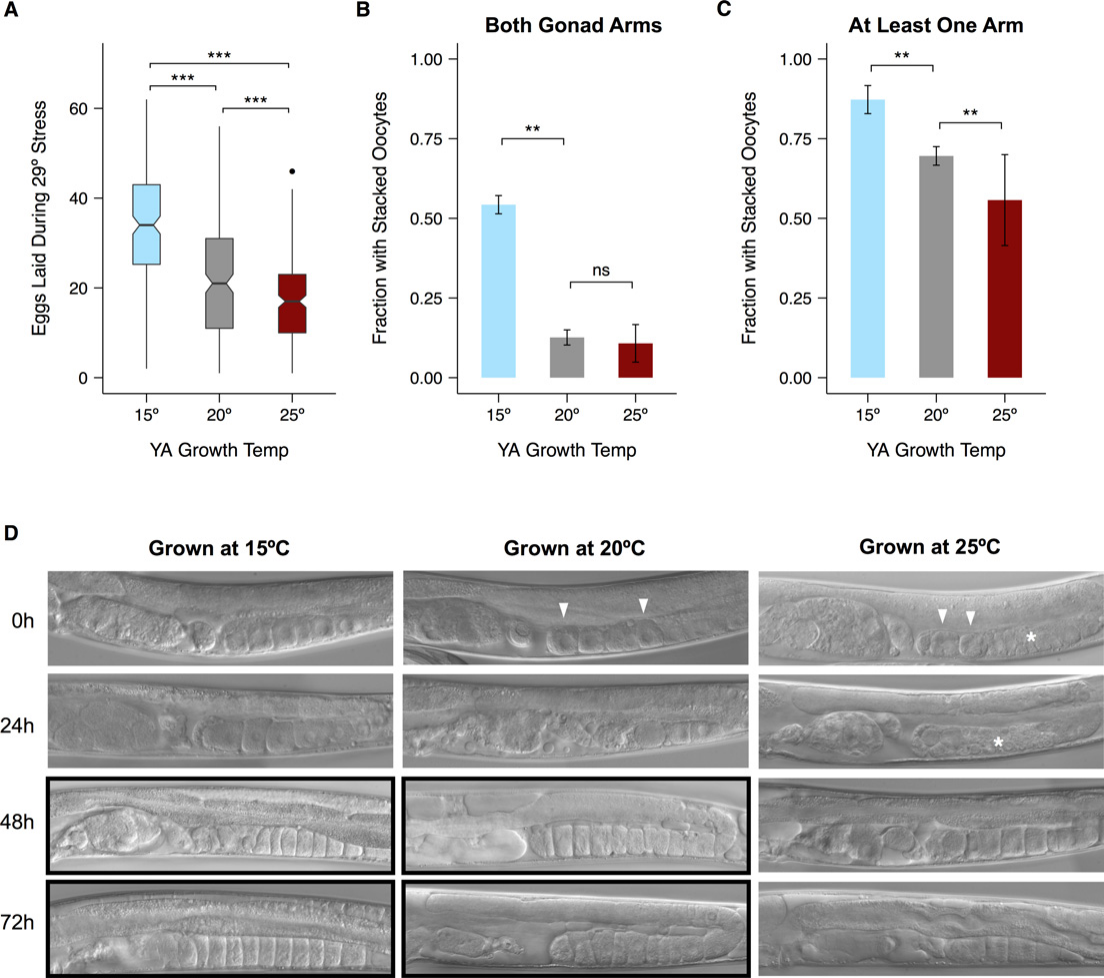
Maternal growth temperature affects damage sustained by the gonad during 29°C heat stress. (A) Number of eggs laid during a 24-hour exposure to 29°C by hermaphrodites raised at different temperatures. Only those worms that laid during the stress are represented. Black lines represent median values. Box hinges represent the first and third quartiles of the data. Whiskers extend to 1.5 × inter-quartile range. (B) Fraction of hermaphrodites with stacked oocytes in both gonad arms after 48 hours of recovery from stress at 29°C. (C) Fraction with stacked oocytes in at least one gonad arm after 48 hours of recovery. (D) Representative images of worms raised at different temperatures during recovery. Images displaying stacked oocytes are boxed in black. White arrowheads point to rounded oocytes and white asterisks highlight disorganized regions of the proximal gonad. Significance levels in (A) were calculated with the Mann-Whitney *U*-test and in (B, C) with the binomial exact test, both with the Bonferroni-Holm correction for multiple testing (**p < 0.01, *** *p* < 0.001, ns: *p* > 0.05). Error bars represent ±1 SEM. See S7 and S9 Figs for individual trials and S3 Table for raw data.

Though worms raised at all three temperatures initiated ovulation at 29°C (Fig 4C), we noticed that those raised at 15°C laid more eggs during stress than their counterparts (Fig 6A, S7 Fig). Observations of individual worms during stress suggested that multiple processes interact to produce this result. More oocytes accumulated in the gonads of worms raised at lower temperatures, while more eggs accumulated in the uteri of worms raised at 25°C (S8 Fig). Because we wanted to understand how worms recover, however, we focused on gonad dynamics after stress.

Visible gonad damage has been most carefully studied during reproductive aging. The distal gonad deteriorates [59] and oocytes in the proximal gonad are more likely to be small in size and to lose contact with neighboring cells, while clusters of unfertilized oocytes accumulate in the uterus [60,61]. We previously documented similar defects in worms raised at 20°C after 24 hours at 29°C [27]. While the most proximal oocytes in healthy, unstressed worms were large and somewhat square, those of recovering worms were often rounded and slightly shrunken, or replaced by clusters of small, round cells. Uteri were full of concretions containing both fertilized and unfertilized oocytes.

Defects of the proximal gonad were less common in worms raised at 15°C, and more common in those raised at 25°C, than in those raised at 20°C (Fig 6D). Later in recovery, one indicator of continued gonad function is the accumulation (stacking) of unfertilized oocytes that results when oocyte production continues in the absence of sufficient sperm to drive ovulation [27,60,62]. We followed stacking in worms recovering from heat stress and chose 48 hours as a representative time point (Fig 6B and 6C, S9 Fig). Worms raised at 15°C were more likely to have stacked oocytes than worms raised at either 20°C or 25°C. This result suggests that worms raised at 15°C are more likely than their counterparts to retain the ability to make healthy oocytes after stress, and thus to make use of the limited supply of functional sperm.

### Maternal experience modulates egg viability at 29°C

Unlike worms raised at 15°C, worms raised at 25°C did not appear to sustain less gonad damage during stress than worms raised at 20°C. However, their embryos were better able to hatch at 29°C (Fig 4A). If this difference results from something intrinsic to oocytes, it may also explain the recovery advantage of 25°C adults entering heat stress (Fig 4B). We previously observed that worms shifted from 20°C to 25°C at young adulthood contained more embryos in the uterus after 24 hours than worms left at 20°C [63]. If worms raised at 25°C also retain embryos longer, their increased age upon laying could account for the hatching advantage we observed.

**Fig 7:**
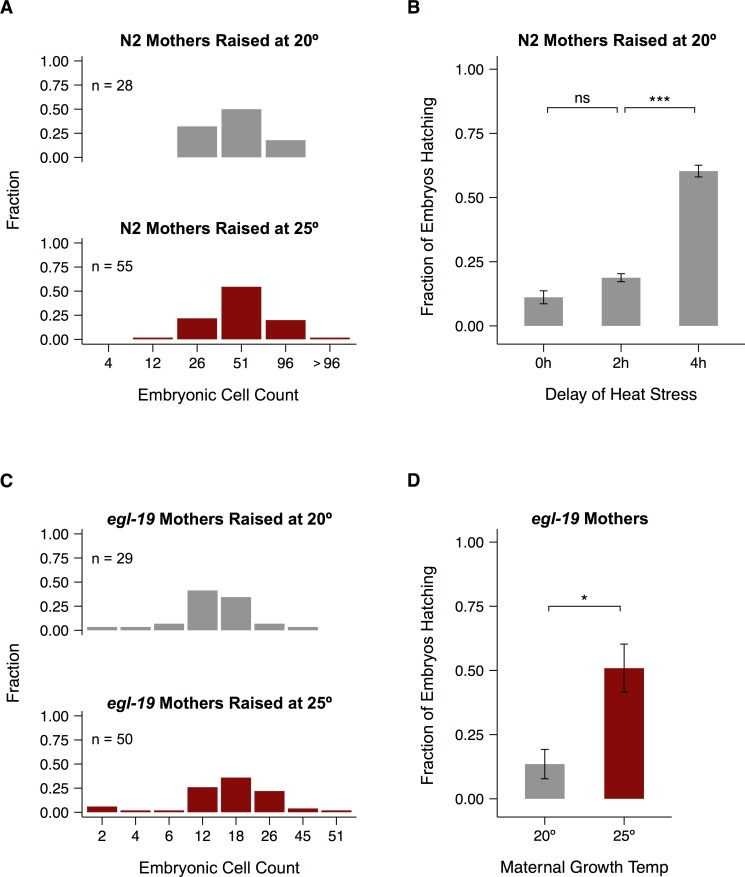
Differences in hatching rates are not due to differences in embryo age. (A, C) Developmental stages of embryos laid by N2 (A) and *egl-19* (C) mothers raised at different temperatures, binned by 30-minute windows (S10 Fig). (B, D) Frequency of hatching during a 24-hour exposure to 29°C for embryos laid by N2 mothers with varying delays in the onset of stress or by *egl-19* mothers raised at different temperatures. Significance levels were calculated in (B) using Welch’s paired *t-*test with Bonferroni-Holm correction and in (D) with the Mann-Whitney *U-*test (*p < 0.05, *** *p* < 0.001, ns: *p* > 0.05). Error bars represent ±1 SEM. See S11 Fig for individual trials and S3 Table for raw data.

To rule out this possibility we counted the cells in freshly laid embryos (see Materials and Methods). The resulting distributions were nearly indistinguishable for embryos laid by mothers raised at 20°C and 25°C (Fig 7A). To further test the sensitivity of hatching success to embryonic development, we allowed embryos laid by mothers raised at 20°C to age for two or four hours before shifting them to 29°C. Two hours of aging did not significantly improve the hatching rate (Fig 7B, S11 Fig), while a four-hour delay increased hatching rates close to the levels observed for mothers raised at 25°C (Fig 4B). After two hours, most embryos were in the midst of the cell migrations that result in dorsal intercalation and epidermal enclosure, though a few had either not yet begun this process or were in the early stages of elongation and morphogenesis (S10 Fig). The dramatic increase in hatching after four hours suggests that the beginning of elongation is the developmental landmark that makes the greatest difference to hatching success.

We also tested whether younger embryos were more sensitive to heat stress using a mutant strain that lays sooner after fertilization than N2 [64,65]. The age distributions were again similar for mothers raised at 20°C and 25°C (Fig 7C), and there was still a significant difference in hatching success (Fig 7D, S11 Fig). In addition, worms raised at 15°C showed a very similar age distribution of embryos at the end of a one-hour egg lay and only minimal increase in hatching success after a three-hour delay of heat stress (S11 Fig), which was developmentally equivalent to the two-hour delay at 20°C. The results reported in this section point to a general mechanism of maternal provisioning for heat stress that increases with increasing growth temperature.

### Transgenerational effects of heat stress

We wondered whether the effects of parental exposure to severe heat stress might also propagate across generations. This pattern would represent a transgenerational form of hormesis, a phenomenon in which a low dose of a stress treatment enhances some subsequent physiological response [66,67]. The hormetic effects of heat shock (brief shifts to near-lethal temperatures) on both thermotolerance and lifespan are well documented in *C*. *elegans*. [7,68–71]. Other demonstrated causes of hormesis include pathogen exposure [72], osmotic stress [73], and excessive dietary glucose [74]. Hormetic effects were transgenerational in the latter two cases.

**Fig 8:**
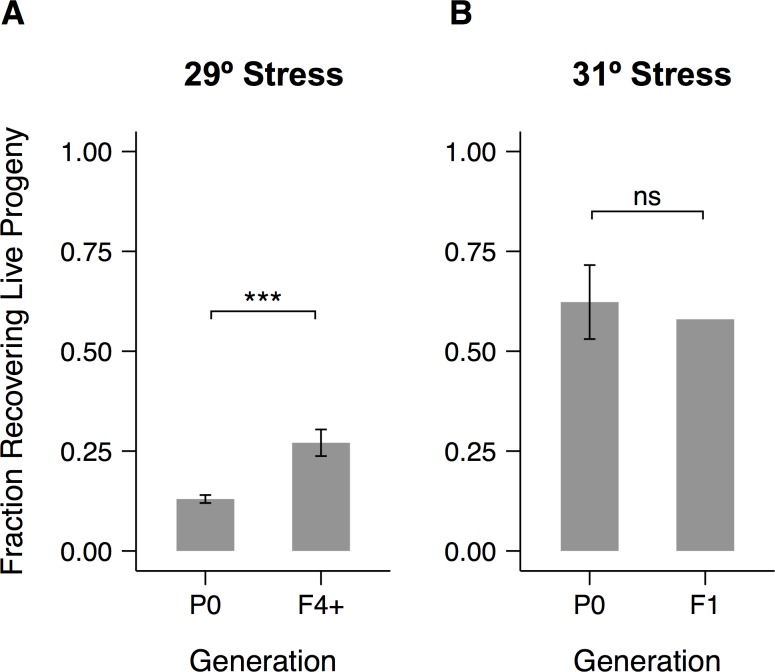
Ancestral exposure to stress improves recovery of fecundity at 29°C but not 31°C. Fractions of adults that recovered live progeny within 5 days after the end of a 24-hour heat stress. (A) Worms stressed at 29°C and their descendants. Embryos were collected by hypochlorite treatment of populations founded by recovering P0 adults. (B) Worms stressed at 31°C and their offspring. F1 embryos were collected by direct hypochlorite treatment of recovering P0 adults. Significance levels were calculated using the binomial exact test (*** *p* < 0.001, ns: p > 0.05). Error bars represent ±1 SEM. See S12 Fig for individual trials and S3 Table for raw data.

We looked for hormesis in the recovery of fecundity after 24 hours of heat stress at 29°C in worms raised at 20°C. Because so few progeny were recovered after this stress treatment, we used individual offspring (Fig 4B) to found populations large enough for synchronization via hypochlorite treatment. The resulting larvae represent at least the F4 generation relative to the original stressed parents. We repeated the same cultivation and stress procedure on these worms and saw a substantial increase in recovery of fecundity (Fig 8A, S12 Fig), comparable to the effects of cultivation at either 15°C or 25°C (Fig 4B). The magnitude of the effect was similar when young adult F1 progeny were individually shifted to a second round of 29°C stress (S12 Fig), suggesting that the effects of ancestral exposure to heat stress do not diminish during the first few generations.

Since recovery is higher after 31°C stress, we isolated F1 larvae by direct hypochlorite treatment of large numbers of recovering adults. When they were subjected to the same stress as their parents, they showed no increase in recovery (Fig 8B), suggesting that baseline reproductive physiology is less malleable under these conditions.

## Discussion

We characterized the effects of cultivation at 15°C, 20°C, and 25°C on a variety of reproductive phenotypes. Exposure to either 15°C or 25°C from L1 arrest through the onset of reproduction is sufficient to affect both lifetime brood size and reproductive performance after exposure to more extreme heat stress, and the effects of some stress treatments can propagate across generations. However, not all aspects of stress response are equally sensitive to prior experience, and different aspects of reproductive physiology respond in different ways. We interpret these complex patterns as evidence that the *C*. *elegans* reproductive system has evolved flexible but coordinated responses to fluctuating and sometimes highly stressful temperatures.

### Scaling and coordination of reproductive development

In keeping with previous studies, we found that the timing of *C*. *elegans* reproductive development is sensitive to temperature. The specific mechanisms controlling the timing of reproductive maturity in *C*. *elegans* are not yet known. However, multiple regulatory systems have been implicated in environmental sensitivity [75,76]. Molts between the larval stages are controlled separately from the stage-specific cell fates within them [77,78], and pathways affecting lifespan at 25°C [79] and 15°C [48,80] can be blocked without changing the time to reproductive maturity. Developmental timing is nevertheless clearly responsive to the environment, since food availability is known to affect progression through all stages of the life cycle [15,24,50,81–85].

Our data suggest that in contrast to the uniform scaling of developmental rate that we saw in *C*. *elegans* (also true in *Drosophila* embryos [86]) that the number of oocytes increased rapidly at all temperatures (S1 and S2 Figs). This appears to harmonize with the limited existing data in *C*. *elegans*. In one mutant strain with delayed oogenesis, the timing of the final molt does not change [87]. Maturing oocytes are known to act together with sperm in regulating their own ovulation [88,89], and the low rate of oocyte accumulation in mutants lacking sperm [88] suggests that this feedback process may extend to oocyte cellularization. Further downstream, egg-laying responds to the presence of sperm [90] and the availability of food [15,91,92]. Because the timing of reproductive development can have profound effects on fitness [87], further research into its control would be worthwhile.

### Lifetime fecundity

By measuring lifetime fecundity in worms exposed to different temperature regimes, we demonstrated thermal effects on physiological processes occurring both before and after the onset of oocyte production (Fig 2). Compared to worms cultivated entirely at 20°C, worms lost some fecundity by developing at 25°C, even more by reproducing at 25°C, and yet more by spending their entire lives at 25°C. Worms lost fecundity to a lesser degree by either developing or reproducing at 15°C, but experienced no further loss by spending the other part of the lifespan at 15°C rather than 20°C. The effects of temperature on sperm production cannot be entirely disentangled from other developmental effects. However, one plausible interpretation of these results is that production and utilization of sperm are balanced at 15°C such that hermaphrodites are able to use most or all of the fertile sperm they produce. At 25°C, by contrast, worms not only seem to produce fewer functional sperm but also appear unable to use all those present at the start of reproduction, perhaps because of continued sperm death, some defect in oogenesis or fertilization, or another yet unidentified mechanism.

Our findings complement the results of previous fecundity experiments in *Caenorhabditis*. *C*. *elegans* hermaphrodites have already been reported to lose fecundity when shifted from 19°C to 25°C during either development or reproduction [58]. However, that study found that the effect of 25°C was stronger during development than reproduction, while we observed the opposite. The discrepancy may either reflect differential sensitivity in worms raised at 19°C and 20°C or some other variation in cultivation conditions. Defects in both spermatogenesis and oogenesis jointly contribute to the loss of hermaphrodite fecundity at 27°C [26]. Reciprocal shifts to cold temperatures have not previously been reported in *C*. *elegans*, but cold had a stronger effect during reproduction in *C*. *briggsae* [57].

### Reproductive performance after stress

Exposure to 15°C or 25°C decreased lifetime fecundity but proved beneficial for several measures of reproductive performance after more extreme heat stress. We focused on two treatments (24 hours of exposure to either 29°C or 31°C) and two phenotypes (embryo hatching and young adult fecundity).

We observed a few broad patterns in treatment responses that cut across growth temperatures. At 31°C, young adults do not lay eggs and embryos laid at cultivation temperatures are unable to hatch. At 29°C, by contrast, young adults lay eggs and embryos laid at cultivation temperatures are sometimes able to hatch, though the rate of hatching depends strongly on the temperature at which their parents grew. Cultivation temperature also influences the ability of young adults to recover fecundity after stress at both 29°C and 31°C, though sperm damage is a major cause of lost fecundity at all temperatures. The brood sizes of all recovering hermaphrodites are very small but can be substantially increased with the addition of fresh sperm.

Our results reveal three important features of the reproductive response to temperature. First, they support the existence of a general strategy by which young adults protectively halt reproductive development once temperatures reach 31°C [27]. Second, they point to a form of physiological plasticity that improves the response to some but not all forms of severe heat stress. The response to 29°C exposure is quite sensitive to cultivation temperature and is improved by ancestral exposure, whereas performance after 31°C exposure is relatively insensitive to cultivation temperature and does not exhibit hormesis. Finally, our results indicate that prior thermal experience improves the response to severe heat stress primarily by influencing the female germline. In addition to reducing the number of fertile sperm, cultivation at 15°C or 25°C does not mitigate the sperm damage caused by exposure to 29°C or 31°C. Instead, we identified a unique change in the female components of the reproductive system at each of these temperatures that likely contributes to the observed increase in recovery after 24 hours at 29°C.

### Protective effects of 15°C cultivation

By examining the gonads of hermaphrodites recovering from heat stress, we showed that cultivation at 15°C mitigates the gonad damage produced by prolonged exposure to 29°C. During stress, worms raised at this temperature laid more eggs. During recovery, they were more likely to retain the ability to produce oocytes in both gonad arms and therefore to make use of the limited supply of surviving sperm. These two phenotypes might reflect separate mechanisms, or they may be part of a unified protective response.

Like other aspects of reproductive timing in *C*. *elegans*, egg-laying is thermally sensitive and has been shown to increase more slowly to a lower peak in worms growing at 16°C than at 20°C or 25°C [45]. Even when heat stress begins before the onset of egg-laying, as in our experiments, prior rates of reproductive development might still influence ovulation during stress. If slower ovulation allows eggs to proceed more smoothly through the heat-stressed gonad, it might counter-intuitively increase the number of eggs laid. This mechanism is consistent with our observation that worms raised at 15°C had more oocytes in the gonad, but fewer eggs in the uterus, than worms raised at 25°C (S7 Fig), and could also explain why most young adults raised at 15°C continued to lay during 30°C stress, in contrast to those raised at 20°C or 25°C (S3 Fig).

One effect of cold exposure is the modulation of membrane fluidity via phospholipid desaturation [93–96]. Recent studies of this pathway have identified a metabolic regulator, PAQ-2, that is essential for successful growth at 15°C [97,98]. If heightened membrane fluidity persists into heat stress, it might offer some physical protection from the damage caused by continued ovulation. However, the persistence of protective effects through several days of recovery suggests instead that cultivation at 15°C affects the ongoing proliferation and renewal of the germ line. Researchers are increasingly looking beyond lipid saturation for other cold tolerance mechanisms, such as induction of small and large heat shock proteins and persistent upregulation of chromatin-modifying genes, that seem more likely to act in this way [99].

### Protective effects of 25°C cultivation

In contrast to cultivation at 15°C, cultivation at 25°C did not visibly decrease gonad damage after 24 hours at 29°C. Instead, it improved the ability of embryos to hatch during this stress and the ability of young adults to recover fecundity afterwards. We established that this hatching advantage is not due to any difference in developmental age. Two possibilities remain—either there is some other physical difference in the embryos or the oocytes themselves are somehow prepared by their mothers to endure future heat stress. At this point there is no evidence to rule out either explanation, and existing research suggests that both are at least possible in *C*. *elegans*.

Poor maternal nutrition has been found to increase the size of *C*. *elegans* embryos [14], and their size and shape may also change at extreme temperatures to compensate for changes in respiration [31]. Phospholipid modulation is also known to be important for growth at 25°C [100]. The heat shock response is induced by a wide variety of stresses [17,101] and may conceivably be primed at 25°C, though evidence of its activity between 25°C and 27°C is scant [102]. Caveolins may also act as heat protectants that could conceivably be maternally regulated. Overexpression of CAV-1 in *C*. *elegans* protected against the loss of fecundity caused by heat shock, and overexpression of CAV-2 increased both the rate and yield of egg-laying [103].

If the same mechanism responsible for the hatching advantage is also responsible for the increase in recovery, it must persist long after the end of heat stress. Again, there is circumstantial evidence to suggest that such long-term persistence is possible in worms. The same maternal nutrient deprivation that increased embryo size also had effects on the fecundity of progeny [14]. In addition, endogenous RNA interference has recently been shown to maintain changes in expression of certain genes for at least two generations after a single generation of exposure to 25°C [104].

## Conclusions

Individual and parental thermal experience modulates multiple components of the reproductive physiology of *C*. *elegans*. Spermatogenesis, oogenesis, and embryogenesis all respond to cultivation temperature in ways that affect recovery from severe heat stress. These processes, and the reproductive outcomes they constitute, nevertheless vary in their sensitivity to prior experience. Such effects, observed under cultivation regimes that are considerably more stable than natural environments, point to complex evolved responses that help worms cope with variable and often stressful temperatures in the wild.

## Materials and Methods

### Preparation of Worm Plates

Most experiments used 60 mm non-vented petri dishes filled with 10 mL of NGM agar; hormesis experiments at 31°C (see below) used 100 mm plates with 30 mL agar [105]. Plates were kept at least a day before use, their lids were shaken to remove moisture, and they were allowed to dry during seeding near an open Bunsen burner. We maintained OP50 at 4°C on streak plates and in liquid culture. Fresh cultures were made monthly by inoculating single colonies into 10 mL of LB broth and incubating overnight with shaking at 37°C. Plates used to maintain worm stocks were seeded with ~50 mL of OP50 culture and left for at least a day to create a large, thick spot of food. To seed a bacterial lawn for plating arrested L1 larvae, we used a sterilized glass rod to spread 100 mL of OP50 culture. For heat stress experiments and brood size experiments, we spotted 5 uL of OP50 culture, diluted between 1:100 and 1:500 in LB broth to seed a thin lawn that could be easily observed under the dissecting scope during recovery. For embryo collection, we seeded a slightly larger and thicker lawn with 10 uL undiluted OP50 culture, using the pipette tip to elongate the drop to facilitate counting later. All plates were left overnight at either 20°C or room temperature after seeding.

### Worm Strains and Maintenance

We used the Bristol N2 strain of *C*. *elegans* and DA695 *egl-19 (ad695)* IV [64,65]. Synchronized cultures of L1 larvae were prepared by hypochlorite treatment of population plates with many gravid hermaphrodites. We were careful to use populations that had not been starved or otherwise stressed for at least the two previous rounds of chunking (3–4 generations). Liberated eggs were allowed to hatch in M9 buffer, which was left at 20°C on a rotisserie for 15–20 hours. To ensure quick and uniform contact with food, we transferred arrested L1 larvae in 5 uL drops to lawn plates using sterile technique. We noted the time that all drops were dry and counted this as 0 hours post L1 arrest. Unless otherwise noted, all worms used for our experiments were plated as arrested L1 larvae at a density of 40 to 70 individuals per plate. We maintained all strains at 20°C and shifted synchronized populations to other cultivation temperatures immediately after plating.

### Temperature Conditions

We cultivated worms at 15°C, 20°C, and 25°C and performed heat stress experiments at 27°–32°C. All temperatures were maintained either in large Percival incubators or in climate-controlled growth chambers. To ensure temperature consistency across experiments performed at different times in different locations, we used a combination of VWR recording thermometers and SmartButton data loggers from ACR Systems. The instruments were periodically calibrated to one another by leaving them together overnight at 15°C, 20°C, or 25°C. When left undisturbed, all incubators and growth chambers maintained temperature within a range of 0.5°C. For heat stress experiments, the recorded range could be wider due to the fluctuations involved in opening and closing the doors. We excluded all experiments in which the temperature varied by more than 1°C over 24 hours.

### Microscopy and Image Analysis

Most of the phenotypes reported here can be counted under a dissecting microscope. When closer examination was necessary, we used a Leica DM5000B compound microscope fitted with a Retiga 2000R camera. To stitch together individual images, we used the MosaicJ plugin [106] with ImageJ software. To view adult worms under the compound scope, we mounted them on agarose pads by picking individuals into a drop of 100 mM sodium azide.

### Developmental Timing

We examined synchronized populations of worms under the compound microscope around the time of the L4–adult molt. Every two hours, we mounted 20 to 30 individuals and observed the morphology of the mouth, cuticle, and vulva. We counted worms as adults when the buccal plug was absent, the mouth open, and the vulva everted from the body wall [107]. Cuticular alae were seen to form before the other milestones, so their presence was used only as corroborating evidence. Occasionally we observed a worm in the process of molting, with vulva everted and mouth open but larval cuticle still loosely attached to the head; such individuals were also counted as adults.

During the first few hours after the L4–adult molt, spermatids become more distinct and cluster at the proximal end of the gonad. Distal to the spermatids, the first oocyte initially appears as an indistinctly grainy region and then completes cellularization [108] to display a uniform cytoplasm and clear nucleus. For individuals scored as adults, we also counted the number of oocytes in the gonad and the number of embryos in the uterus. Oocytes were only counted when they were large enough to span the lumen of the gonad.

Population density was controlled extremely carefully for these experiments so that all worms examined grew up on plates containing between 45 and 55 individuals. A fresh plate was used for each time point. To minimize variation between trials, experiments were repeated in the same incubator within a period of a few months. As described above (Experimental Rationale), we considered worms “young adults” when they produced their first oocyte. Based on the census results, we selected a target time for each temperature— 39h at 25°C, 50h at 20°C, 90h at 15°C—at which approximately 50% of the population reached this landmark. In performing experiments over several years, however, we observed that developmental timing, particularly at lower temperatures, was sensitive to small fluctuations in growth temperature and population density. When singling young adult worms for all other experiments, we therefore used population morphology rather than absolute time to identify the target stage. For each growth temperature, we started experiments within the following ranges of time after plating: 15°C between 90 and 94 hours, 20°C between 48 and 51 hours, 25°C between 39 and 40 hours.

### Brood Size

For all brood size experiments, we grew worms in synchronized populations at 15°C, 20°C, or 25°C and singled them as young adults. Depending on the experiments performed, these plates were either returned to the original cultivation temperature or shifted to a different one. Adults reproducing at 20°C or 25°C were transferred to new plates every day, and those at 15°C were transferred every other day. We kept plates at the reproduction temperature long enough for eggs to hatch and then transferred them to 15°C to slow larval growth and facilitate counting. Larvae were counted at the L3 to L4 stage, before they began producing their own progeny. When an adult had produced no more than one embryo during two successive counting periods, we stopped looking for new progeny. We censored worms that left the agar or burrowed unless they had produced <5 embryos during the preceding counting period.

### Heat Stress and Recovery

For each heat stress experiment, 25 or 50 worms were singled as young adults. For the duration of the experiment, plates were kept in stacks of five, rubber-banded together with agar side up, inside a plastic shoebox. At the end of 24 hours, the box was removed from the stress temperature and the eggs laid by each worm during stress were immediately counted. Worms were allowed to recover at 20°C for at least 8 days. We counted the total number of live progeny at least once before any had a chance to begin reproduction (on the third or fourth day of recovery, depending on the experiment). To confirm brood sizes and detect any newly recovered worms, we repeated the count on the fifth day to yield the data reported above. We also kept the plates an additional three or four days and recorded whether they had expanding populations. Most worms that eventually recovered did so by the fifth day, but occasionally we observed new progeny during this final check, and some progeny also proved infertile. Worms that died before the end of heat stress were censored from the experiment.

### Mating Rescue

Mating rescue experiments were performed exactly as above, except that three unstressed young adult males were added to half the plates immediately after the end of heat stress. We segregated these males the previous day, selecting late L4 or early adults that crawled vigorously and had normal tail morphology.

### Effects of Stress on the Gonad

Worms observed on the compound microscope during or after heat stress were shifted as synchronized small populations. For the gonad census during stress (S8 Fig), we counted oocytes in the gonad and eggs in the uterus in 20 individuals from these populations at the start of heat stress and at 2, 4, 6, 8, 10, 12, and 24 hours thereafter, using a fresh plate for each time point. For observations of gonad damage during recovery (Fig 6D), we mounted 20–30 worms at the end of heat stress and at 24, 48, and 72 hours thereafter. For quantification of stacking (Fig 6B and 6C), we removed all L4 animals on the growth plate before shifting to 29°C.

### Embryo Hatching

To collect a population of embryos for heat stress, we transferred 12–18 gravid hermaphrodites from a synchronized population plate to a fresh plate, being careful to immediately remove any embryos or larvae transferred along with the adults. Adults were allowed to lay for 1 hour and the plates were kept at their original cultivation temperature. Worms generally required 10–15 minutes to recover from the transfer before resuming laying. After the end of the laying period, we removed the adults and shifted the embryos to a stress temperature for 24 hours. For some experiments, we allowed embryos to age at the maternal cultivation temperature for 2–4 hours between the removal of the adults and the onset of the heat stress.

### Embryo Staging

To identify the stage at which eggs were laid, we first collected embryos as described above. To slow additional cell divisions, we kept embryos chilled whenever possible during slide preparation. We used ice-cold M9 to rinse embryos and food off the plates into 1.5 mL tubes, then removed excess liquid after spinning them in a microcentrifuge at 4°C for 1 minute at a speed of 0.5 × *g*. The remaining volume (~10 uL) was spotted directly onto a chilled agar pad. Prepared slides were then photographed on the compound scope, and we used the photographs to identify the number of cells present in each embryo. Slide preparation typically took around 20 minutes, and imaging took an additional 30 minutes.

Classic lineage studies established that embryonic cell counts do not increase uniformly over time [109,110]. To determine how to compare cell counts across treatments, we consulted recent lineage studies that refine these measurements [30,111–114]. Richards et al. [30] timed each cell division through the onset of morphogenesis in multiple embryos and normalized the resulting mean times to the canonical 20° lineage reported by Sulston [110]. We used a plot derived from their published data (S10 Fig) to select 30-minute intervals of developmental time, counting from the first cleavage, for binning our cell counts. These times fall during plateaus in the curve, representing periods between rounds of cell division.

### Hormesis

We used several different methods to test whether the descendants of stressed worms were themselves better able to recover from the same heat stress experienced by their ancestors. After heat stress at 31°C, we were able to generate a synchronized population of progeny by hypochlorite treatment of adults recovering from heat stress. We grew these worms on larger plates at equivalent population density, shifted them to the stress temperature when most were young adults, and then collected them for hypochlorite treatment 22–26 hours after the end of the stress. A starting population of ~1500 stressed individuals yielded only 62 larvae, of which 50 matured on schedule. We singled the latter group to perform another heat stress experiment as described above.

Since the rate of recovery is even lower after 29°C than 31°C stress, we did not attempt this procedure with worms stressed at 29°C. Instead, we selected 3–4 individual progeny per experiment and propagated them separately for several generations until there were enough gravid adults in each population for hypochlorite treatment. The resulting synchronized populations, representing at least the F4 generation relative to the original stressed P0 individuals, were used to repeat the heat stress protocol.

To test whether hormesis was stronger in the direct progeny of stressed worms, we also collected 31 individual progeny from 167 individual stressed adults. These individuals hatched between 60 and 144 hours after the end of heat stress. As each one reached the young adult stage, we shifted it individually to the same heat stress conditions experienced by its parents.

## Supporting Information

S1 Fig Timing of reproductive maturity. Each panel shows a single trial. Times are given in hours since plating of arrested L1 larvae on food. Solid lines indicate the fraction of worms that have completed the final molt, and dashed lines the fraction that have formed at least one oocyte. See S1 Table for raw data.
(PDF)

S2 Fig Onset of oocyte production. Histograms represent the number of cellularized oocytes at different time points in both gonad arms for worms raised at (A) 15°C, (B) 20°C, or (C) 25°C. Times are given in hours since plating of arrested L1 larvae on food. See S1 Table for raw data.
(PDF)

S3 Fig Reproductive performance across a range of heat stress temperatures. Blue, gray, and red lines represent worms cultivated at 15°C, 20°C, or 25°C, respectively. (A) Fraction of eggs laid by mothers raised at each temperature that hatched during a 24-hour exposure to the stress temperature. (B) Fraction of young adults raised at each temperature that recovered live progeny within 5 days after the end of a 24-hour exposure to the stress temperature. (C) Fraction of those same young adults that laid eggs during the stress period. Error bars represent ±1 SEM. See S3 Table for raw data.
(PDF)

S4 Fig Recovery of fecundity after heat stress for N2 and various wild isolate strains of *C*. *elegans* raised at 20°C. We compared the ability of various wild isolate strains of *C*. *elegans* and N2 to recover fecundity across a range of heat stress temperatures. Strains were selected to sample the latitudinal diversity of *C*. *elegans* habitats and to be genetically divergent from N2. The results fell into three patterns. (A) CB4856 (Hawaii) and JU440 (Beauchene, France) have recovery of fecundity curves that are similar to N2. The three strains all have a "bump" in recovery at 31°C. (B) The curves of MY1 (Lingen, Germany) and MY2 (Roxel, Germany) are similar to N2 but the "bump" appears shifted one-half degree warmer to 31.5°C. (C) Surprisingly, JU258 (Madeira) did not recover fecundity after heat stress very well at any of the temperatures we tested. The N2 recovery data are plotted for comparison and are the same in all three panels. Numbers of independent trials and the raw data are provided in S4 Table.
(PDF)

S5 Fig Reproductive performance after 29°C and 31°C heat stress. (A, B) Individual trials (each with n = 25) for the recovery results reported in Fig 5A and 5B. (C) Brood sizes of recovered individuals raised at 15°C (blue) or 25°C (red) increased when fresh males were added during recovery from stress at 29°C. Black lines represent median values. Box hinges represent the first and third quartiles of the data. Each whisker extends to the furthest data point within 1.5 × *IQR* (inter-quartile range) of its nearest hinge.
(PDF)

S6 Fig Severe heat stress causes extensive sperm damage. (A, B) Individual trials (each with n = 25) for the recovery results reported in Fig 5A and 5B. (C) Brood sizes of recovered individuals raised at 15°C (blue) or 25°C (red) increased when fresh males were added during recovery.
(PDF)

S7 Fig Egg-laying during 29°C heat stress. Individual trials (each with n = 25 or n = 50) for the egg-laying results reported in Fig 6A. Each point represents the number of eggs laid by one worm. See S3 Table for raw data.
(PDF)

S8 Fig Gonad dynamics during 29°C heat stress. Lines show the accumulation of (A) oocytes in the gonad and (B) eggs in the uterus during heat stress at 29°C. See S3 Table for raw data.
(PDF)

S9 Fig Gonad damage after 29°C heat stress. (A) Stacking over time in each gonad arm during recovery from 29°C heat stress (30 ≤ n ≤ 38 for each time point). (B, C) Individual trials (each with 30 ≤ n ≤ 41) for the stacking results reported in Fig 6. See S3 Table for raw data.
(PDF)

S10 Fig Embryonic cell counts over time. (A) Data from [30] showing the increase in embryonic cell count as a function of time. (B) The same data plotted over a longer interval, with annotations showing important cell movements drawn from [110,114]. See S3 Table for raw data.
(PDF)

S11 Fig Effects of maternal cultivation temperature on hatching. (A, B) Individual trials (each with 63 ≤ n ≤ 260) for the hatching data reported in Fig 7B and 7D. Embryos laid during the same collection period are connected with dashed lines. (C) Cell counts of embryos laid by mothers raised at 15°C. (D) A 3-hour delay of heat stress only marginally improved the hatching rate of embryos laid by mothers raised at 15°C. See S3 Table for raw data.
(PDF)

S12 Fig Hormesis in the descendants of worms raised at 20°C and stressed at 29°C. (A) Individual experiments (each with n = 50) reported collectively in Fig 8A. (B) Results of a separate experiment in which the F1 offspring of stressed parents were themselves stressed at 29°C (binomial exact test *p* = 0.044). See S3 Table for raw data.
(PDF)

S1 Table Raw data for developmental timing at 15°C, 20°C, and 25°C. (XLSX)

S2 Table Raw data for brood size experiments. (XLSX)

S3 Table Raw data for recovery from heat stress. (XLSX)

S4 Table Raw data for recovery in wild isolate strains. (XLSX)
