# Supplementary material for: Experience Modulates the Reproductive Response to Heat Stress in C. elegans via Multiple Physiological Processes
Source: PLoS One. 2015 Dec 29;10(12):e0145925. doi: 10.1371/journal.pone.0145925 (PMC4699941; doi:10.1371/journal.pone.0145925)
Supplement: S1 Fig — Each panel shows a single trial. Times are given in hours since plating of arrested L1 larvae on food. Solid lines indicate the fraction of worms that have completed the final molt, and dashed lines the fraction that have formed at least one oocyte. See S1 Table for raw data. (PDF) [file pone.0145925.s001.pdf]

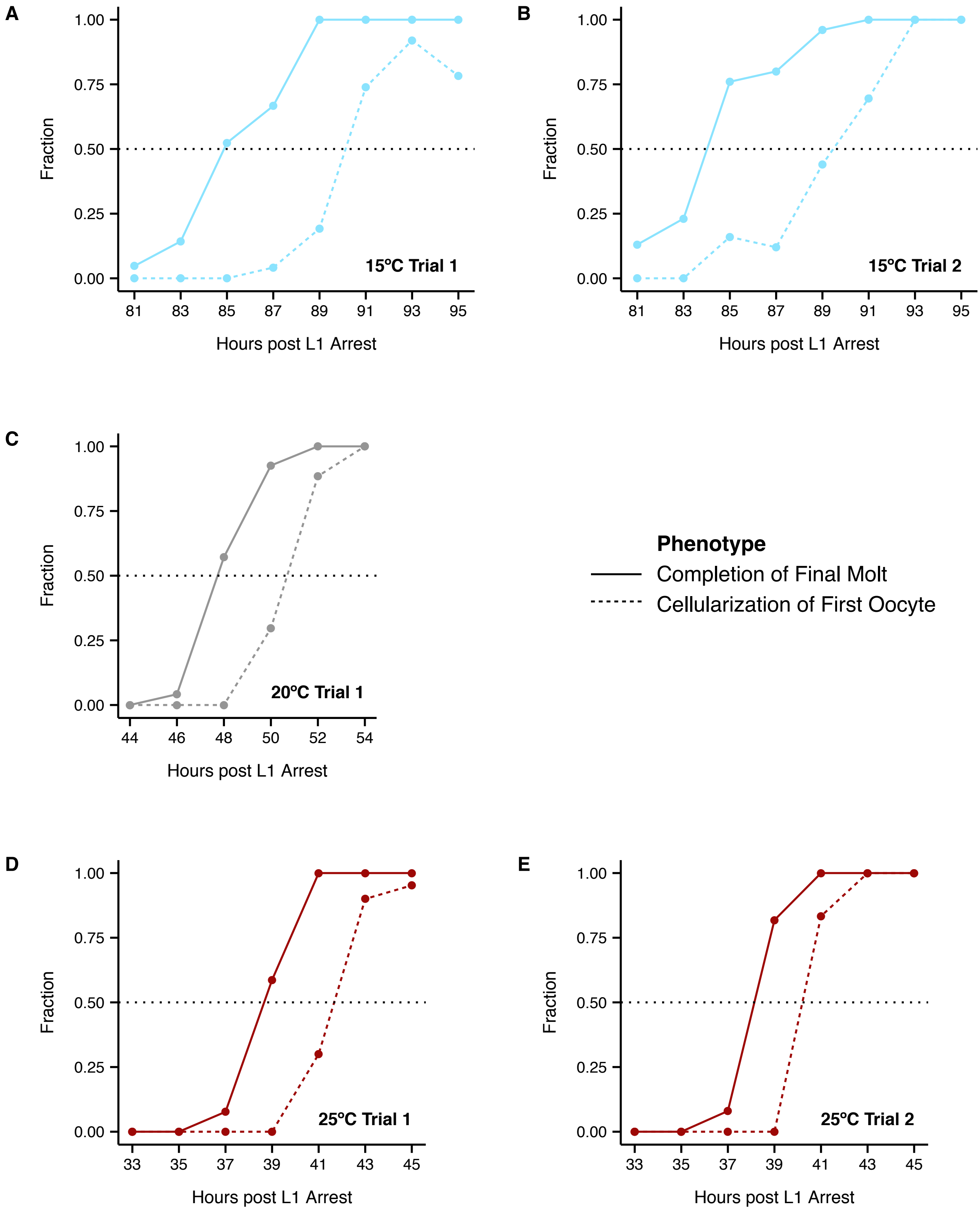

S1 Fig. Timing of reproductive maturity. Each panel shows a single trial. Times are given in hours since plating of arrested L1 larvae on food. Solid lines indicate the fraction of worms that have completed the final molt, and dashed lines the fraction that have formed at least one oocyte. See S1 Table for raw data.
