# Supplementary material for: Experience Modulates the Reproductive Response to Heat Stress in C. elegans via Multiple Physiological Processes
Source: PLoS One. 2015 Dec 29;10(12):e0145925. doi: 10.1371/journal.pone.0145925 (PMC4699941; doi:10.1371/journal.pone.0145925)
Supplement: S2 Fig — Histograms represent the number of cellularized oocytes at different time points in both gonad arms for worms raised at (A) 15°C, (B) 20°C, or (C) 25°C. Times are given in hours since plating of arrested L1 larvae on food. See S1 Table for raw data. (PDF) [file pone.0145925.s002.pdf]

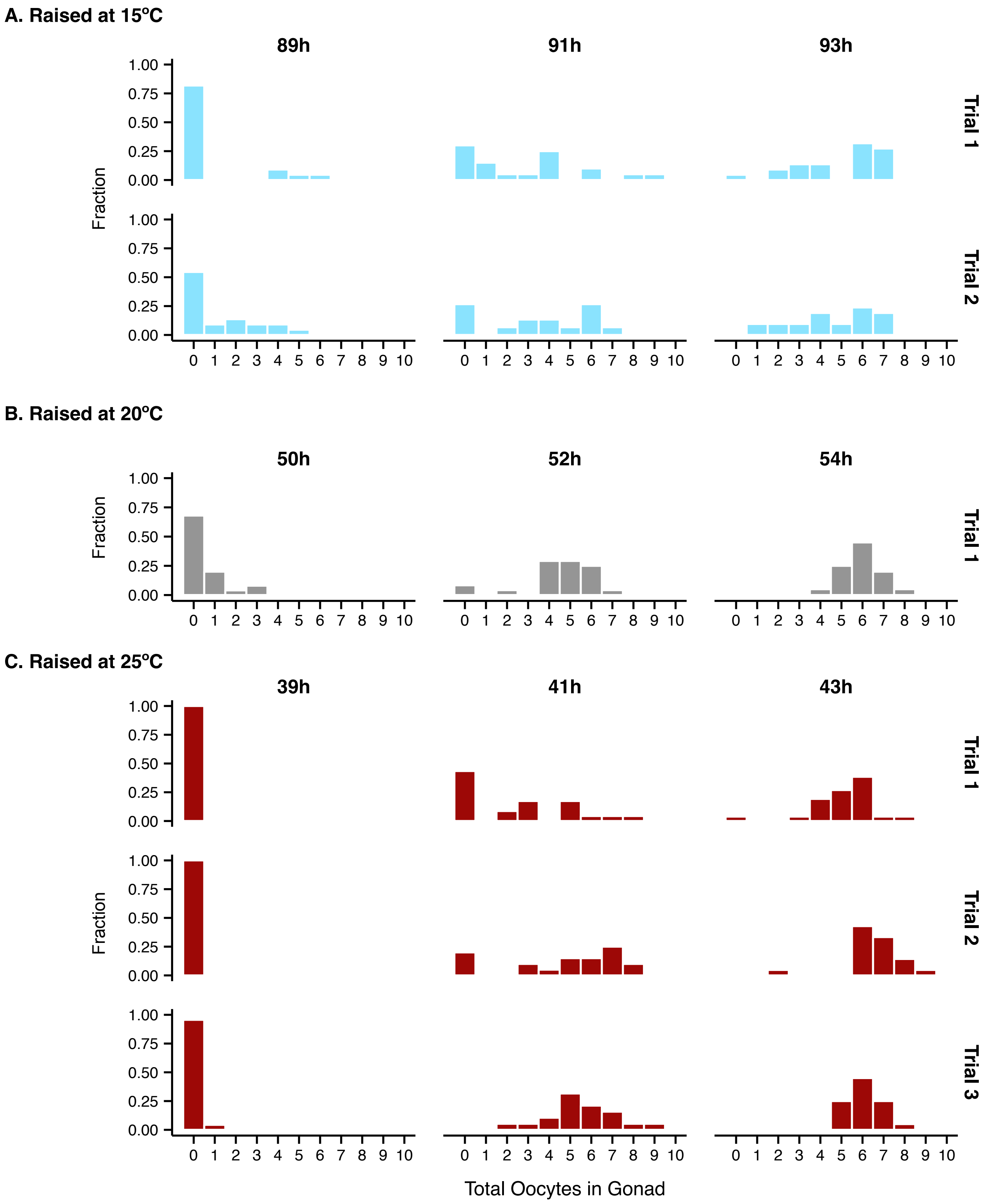

S2 Fig. Onset of oocyte production. Histograms represent the number of cellularized oocytes at different time points in both gonad arms for worms raised at (A) 15ºC, (B) 20ºC, or (C) 25ºC. Times are given in hours since plating of arrested L1 larvae on food. See S1 Table for raw data.
