# Supplementary material for: Experience Modulates the Reproductive Response to Heat Stress in C. elegans via Multiple Physiological Processes
Source: PLoS One. 2015 Dec 29;10(12):e0145925. doi: 10.1371/journal.pone.0145925 (PMC4699941; doi:10.1371/journal.pone.0145925)
Supplement: S3 Fig — Blue, gray, and red lines represent worms cultivated at 15°C, 20°C, or 25°C, respectively. (A) Fraction of eggs laid by mothers raised at each temperature that hatched during a 24-hour exposure to the stress temperature. (B) Fraction of young adults raised at each temperature that recovered live progeny within 5 days after the end of a 24-hour exposure to the stress temperature. (C) Fraction of those same young adults that laid eggs during the stress period. Error bars represent ±1 SEM. See S3 Table for raw data. (PDF) [file pone.0145925.s003.pdf]

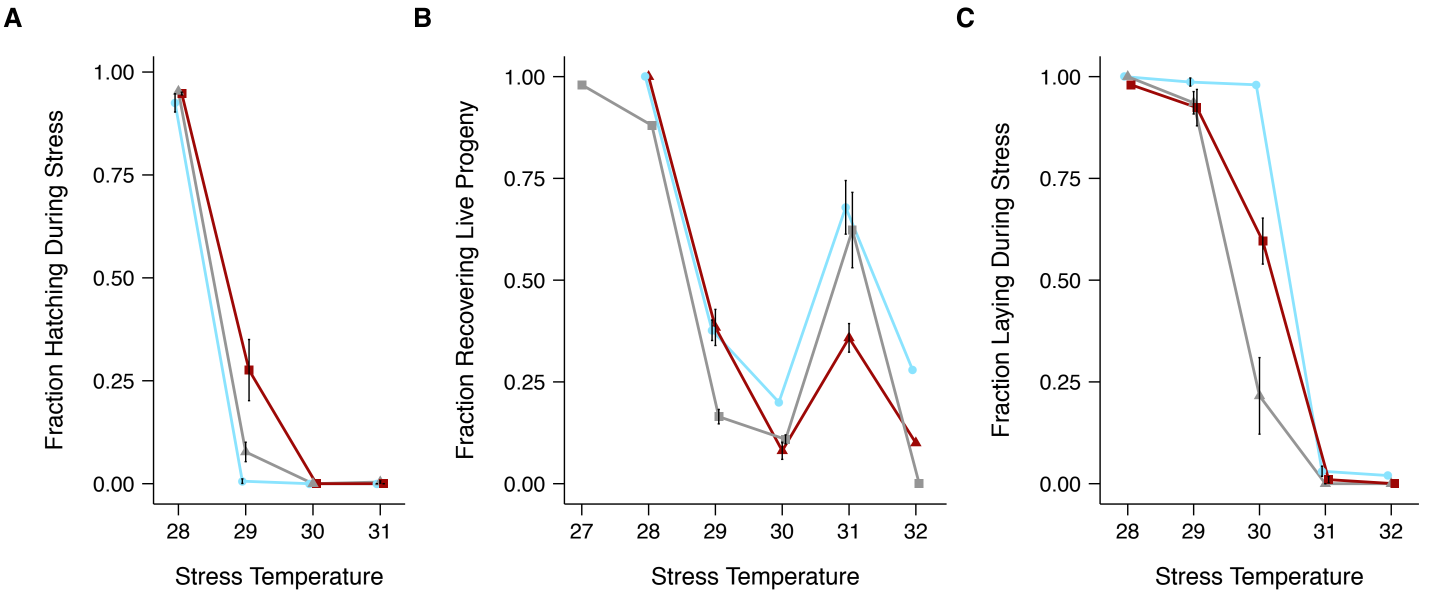

S3 Fig. Reproductive performance across a range of heat stress temperatures. Blue, gray, and red lines represent worms cultivated at 15ºC, 20ºC, or 25ºC, respectively. (A) Fraction of eggs laid by mothers raised at each temperature that hatched during a 24-hour exposure to the stress temperature. (B) Fraction of young adults raised at each temperature that recovered live progeny within 5 days after the end of a 24-hour exposure to the stress temperature. (C) Fraction of those same young adults that laid eggs during the stress period. Error bars represent ±1 SEM. See S3 Table for raw data.
