# Supplementary material for: Experience Modulates the Reproductive Response to Heat Stress in C. elegans via Multiple Physiological Processes
Source: PLoS One. 2015 Dec 29;10(12):e0145925. doi: 10.1371/journal.pone.0145925 (PMC4699941; doi:10.1371/journal.pone.0145925)
Supplement: S4 Fig — We compared the ability of various wild isolate strains of C. elegans and N2 to recover fecundity across a range of heat stress temperatures. Strains were selected to sample the latitudinal diversity of C. elegans habitats and to be genetically divergent from N2. The results fell into three patterns. (A) CB4856 (Hawaii) and JU440 (Beauchene, France) have recovery of fecundity curves that are similar to N2. The three strains all have a "bump" in recovery at 31°C. (B) The curves of MY1 (Lingen, Germany) and MY2 (Roxel, Germany) are similar to N2 but the "bump" appears shifted one-half degree warmer to 31.5°C. (C) Surprisingly, JU258 (Madeira) did not recover fecundity after heat stress very well at any of the temperatures we tested. The N2 recovery data are plotted for comparison and are the same in all three panels. Numbers of independent trials and the raw data are provided in S4 Table. (PDF) [file pone.0145925.s004.pdf]

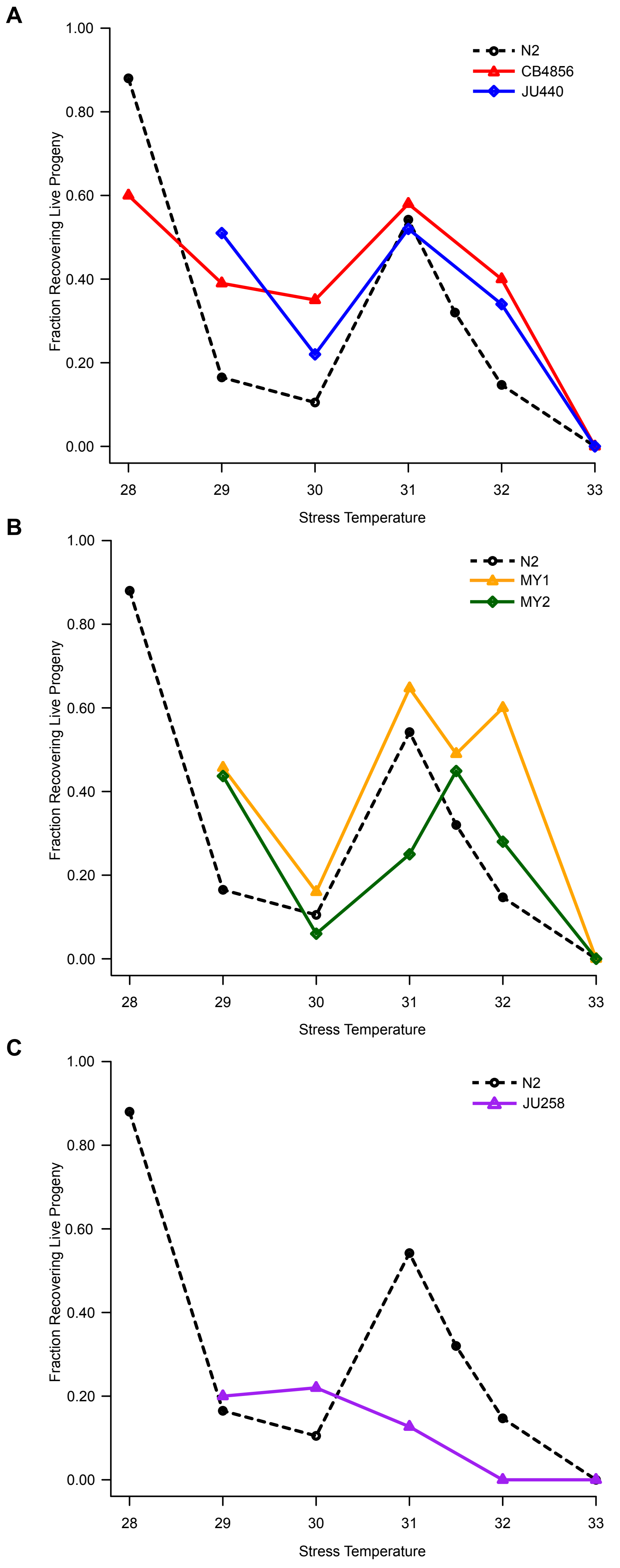

S4 Fig. Recovery of fecundity after heat stress for N2 and various wild isolate strains of *C. elegans* raised at 20°C. We compared the ability of various wild isolate strains of *C. elegans* and N2 to recover fecundity across a range of heat stress temperatures. Strains were selected to sample the latitudinal diversity of *C. elegans* habitats and to be genetically divergent from N2. The results fell into three patterns. (A) CB4856 (Hawaii) and JU440 (Beauchene, France) have recovery of fecundity curves that are similar to N2. The three strains all have a "bump" in recovery at 31°C. (B) The curves of MY1 (Lingen, Germany) and MY2 (Roxel, Germany) are similar to N2 but the "bump" appears shifted one-half degree warmer to 31.5°C. (C) Surprisingly, JU258 (Madeira) did not recover fecundity after heat stress very well at any of the temperatures we tested. The N2 recovery data are plotted for comparison and are the same in all three panels. Numbers of independent trials and the raw data are provided in S4 Table.
