# Supplementary material for: Experience Modulates the Reproductive Response to Heat Stress in C. elegans via Multiple Physiological Processes
Source: PLoS One. 2015 Dec 29;10(12):e0145925. doi: 10.1371/journal.pone.0145925 (PMC4699941; doi:10.1371/journal.pone.0145925)
Supplement: S5 Fig — (A, B) Individual trials (each with n = 25) for the recovery results reported in Fig 5A and 5B. (C) Brood sizes of recovered individuals raised at 15°C (blue) or 25°C (red) increased when fresh males were added during recovery from stress at 29°C. Black lines represent median values. Box hinges represent the first and third quartiles of the data. Each whisker extends to the furthest data point within 1.5 × IQR (inter-quartile range) of its nearest hinge. (PDF) [file pone.0145925.s005.pdf]

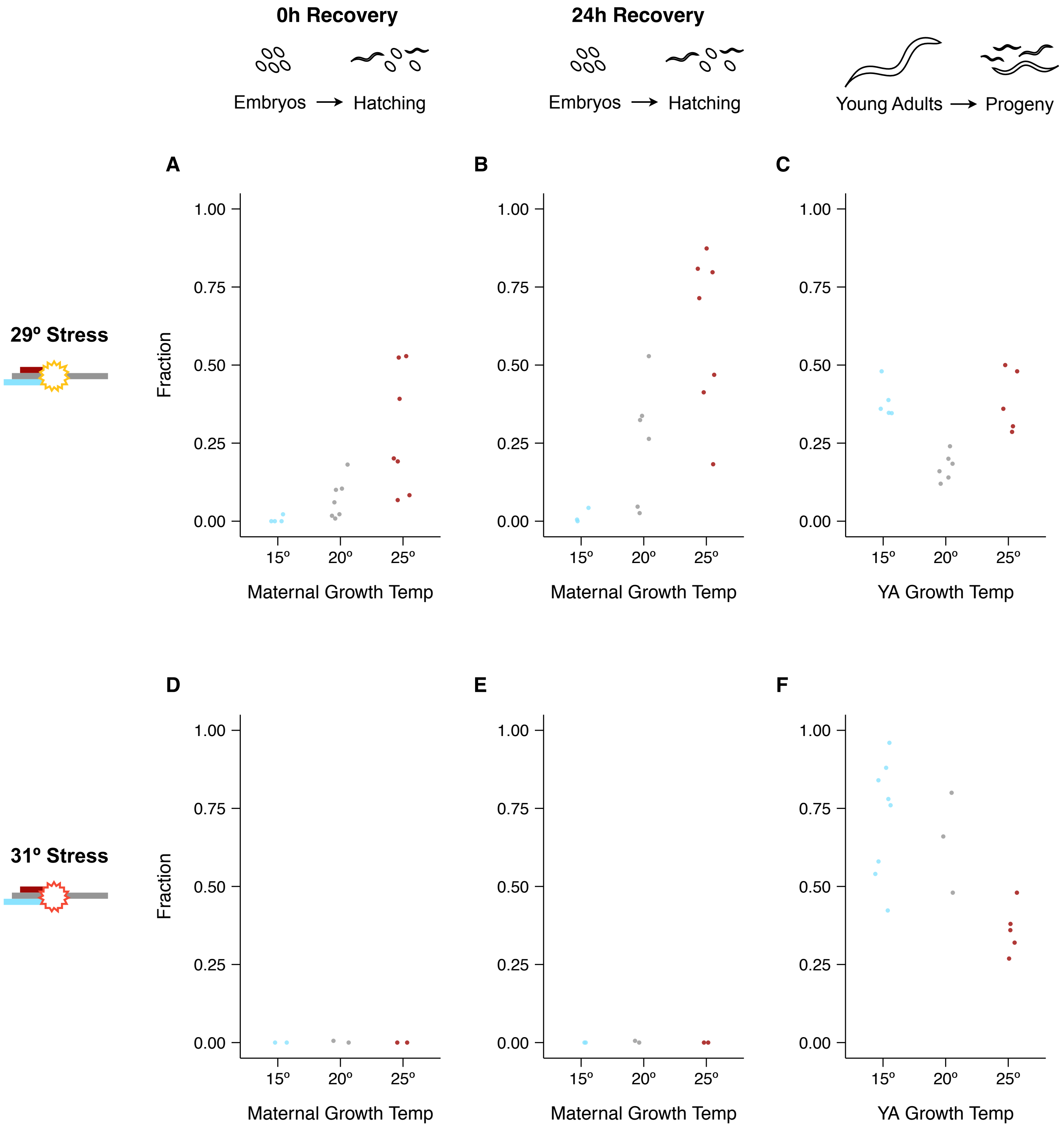

S5 Fig. Reproductive performance after 29ºC and 31ºC heat stress. (A, D) Individual trials (each with 47 ≤ n ≤ 279) for the hatching results reported in Fig. 4A and 4D. (B, E) Hatching fraction increases after 24 hours of recovery from 29ºC but not 31ºC heat stress. (C, F) Individual trials (each with n = 25 or n = 50) for the recovery results reported in Figure 4B and 4E. See S3 Table for raw data.
