# Supplementary material for: Experience Modulates the Reproductive Response to Heat Stress in C. elegans via Multiple Physiological Processes
Source: PLoS One. 2015 Dec 29;10(12):e0145925. doi: 10.1371/journal.pone.0145925 (PMC4699941; doi:10.1371/journal.pone.0145925)
Supplement: S6 Fig — (A, B) Individual trials (each with n = 25) for the recovery results reported in Fig 5A and 5B. (C) Brood sizes of recovered individuals raised at 15°C (blue) or 25°C (red) increased when fresh males were added during recovery. (PDF) [file pone.0145925.s006.pdf]

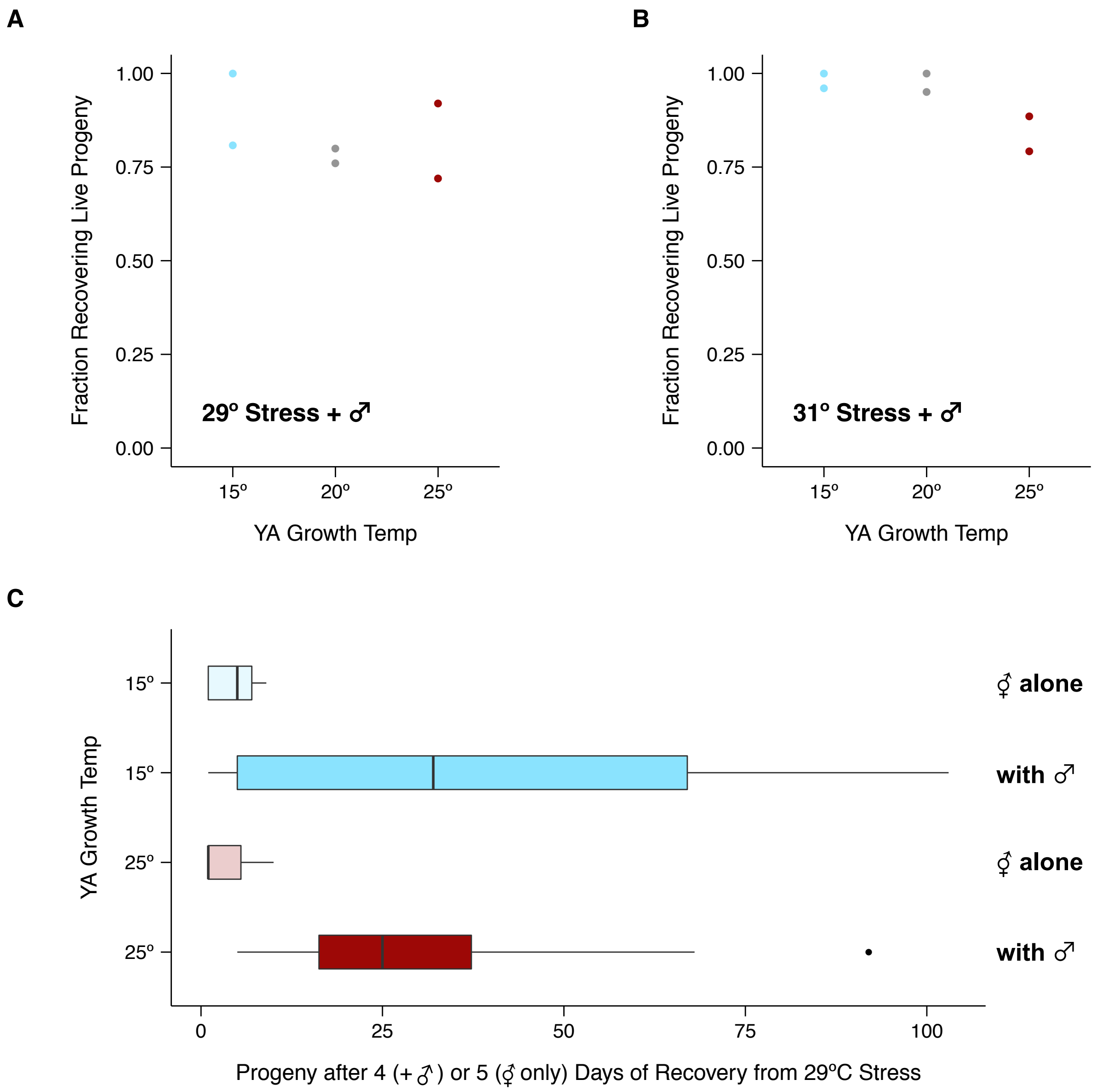

S6 Fig. Severe heat stress causes extensive sperm damage. (A, B) Individual trials (each with n = 25) for the recovery results reported in Fig. 5A and 5B. (C) Brood sizes of recovered individuals raised at 15ºC (blue) or 25ºC (red) increased when fresh males were added during recovery from stress at 29ºC. Black lines represent median values. Box hinges represent the first and third quartiles of the data. Each whisker extends to the furthest data point within 1.5 × *IQR* (inter-quartile range) of its nearest hinge.
