# Supplementary material for: Experience Modulates the Reproductive Response to Heat Stress in C. elegans via Multiple Physiological Processes
Source: PLoS One. 2015 Dec 29;10(12):e0145925. doi: 10.1371/journal.pone.0145925 (PMC4699941; doi:10.1371/journal.pone.0145925)
Supplement: S7 Fig — Individual trials (each with n = 25 or n = 50) for the egg-laying results reported in Fig 6A. Each point represents the number of eggs laid by one worm. See S3 Table for raw data. (PDF) [file pone.0145925.s007.pdf]

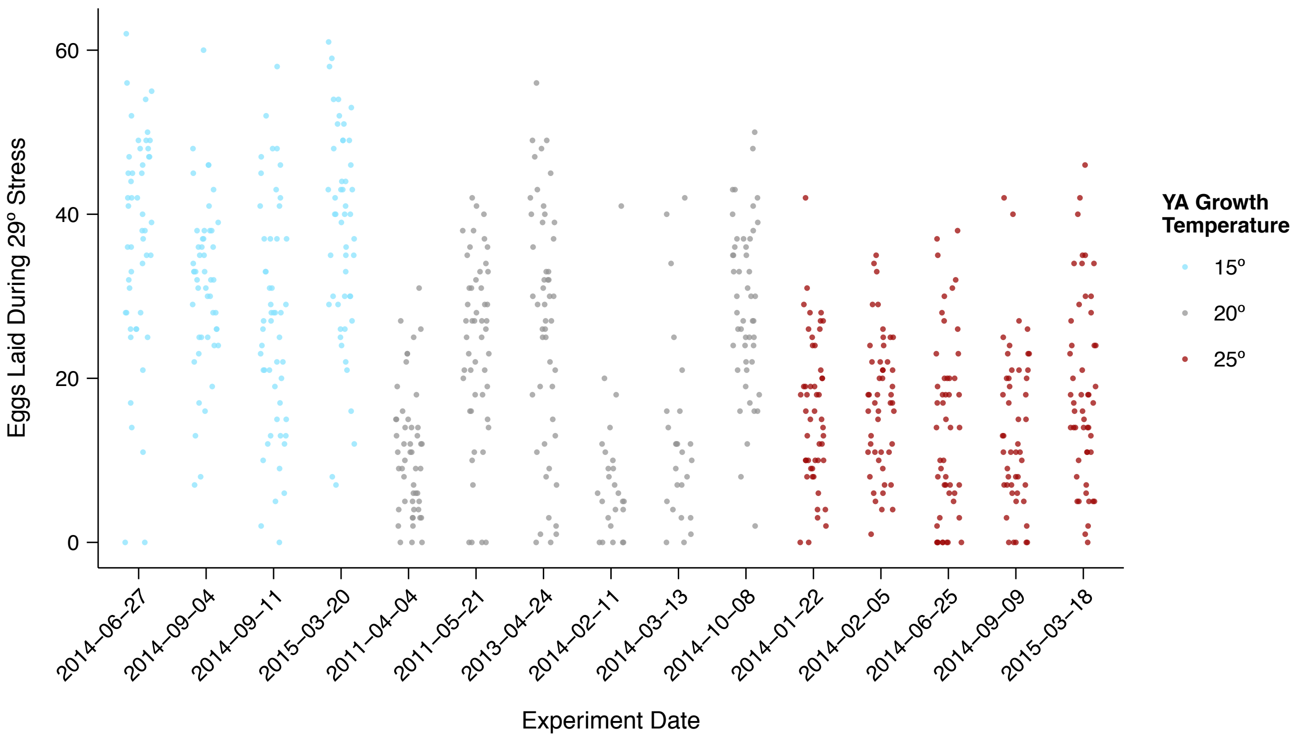

S7 Fig. Egg-laying during 29ºC heat stress. Individual trials (each with n = 25 or n = 50) for the egg-laying results reported in Fig. 6A. Each point represents the number of eggs laid by one worm. See S3 Table for raw data.
