# Supplementary material for: Experience Modulates the Reproductive Response to Heat Stress in C. elegans via Multiple Physiological Processes
Source: PLoS One. 2015 Dec 29;10(12):e0145925. doi: 10.1371/journal.pone.0145925 (PMC4699941; doi:10.1371/journal.pone.0145925)
Supplement: S8 Fig — Lines show the accumulation of (A) oocytes in the gonad and (B) eggs in the uterus during heat stress at 29°C. See S3 Table for raw data. (PDF) [file pone.0145925.s008.pdf]

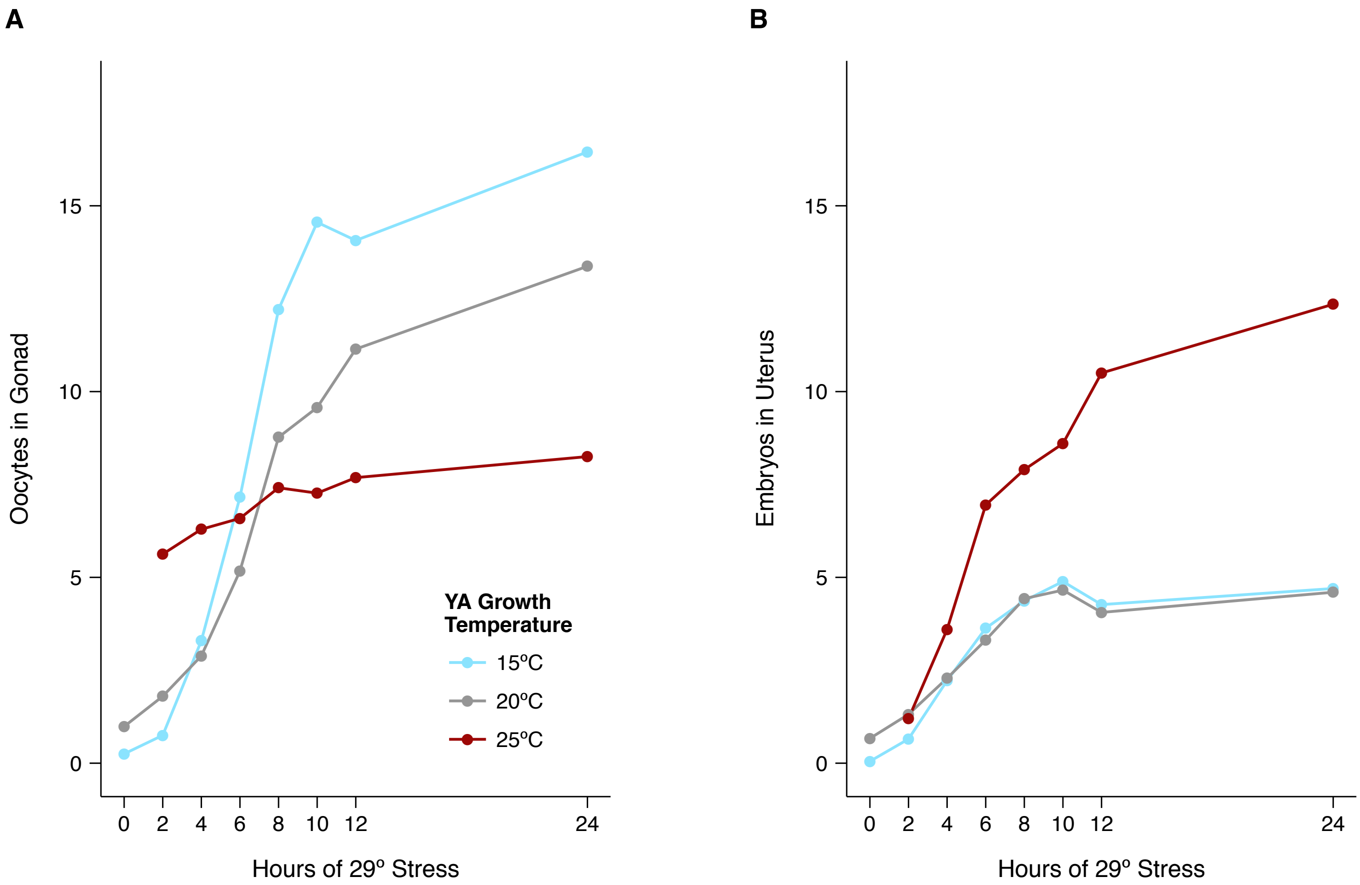

S8 Fig. Gonad dynamics during 29ºC heat stress. Lines show the accumulation of (A) oocytes in the gonad and (B) eggs in the uterus during heat stress at 29ºC. See S3 Table for raw data.
