# Supplementary material for: Experience Modulates the Reproductive Response to Heat Stress in C. elegans via Multiple Physiological Processes
Source: PLoS One. 2015 Dec 29;10(12):e0145925. doi: 10.1371/journal.pone.0145925 (PMC4699941; doi:10.1371/journal.pone.0145925)
Supplement: S9 Fig — (A) Stacking over time in each gonad arm during recovery from 29°C heat stress (30 ≤ n ≤ 38 for each time point). (B, C) Individual trials (each with 30 ≤ n ≤ 41) for the stacking results reported in Fig 6. See S3 Table for raw data. (PDF) [file pone.0145925.s009.pdf]

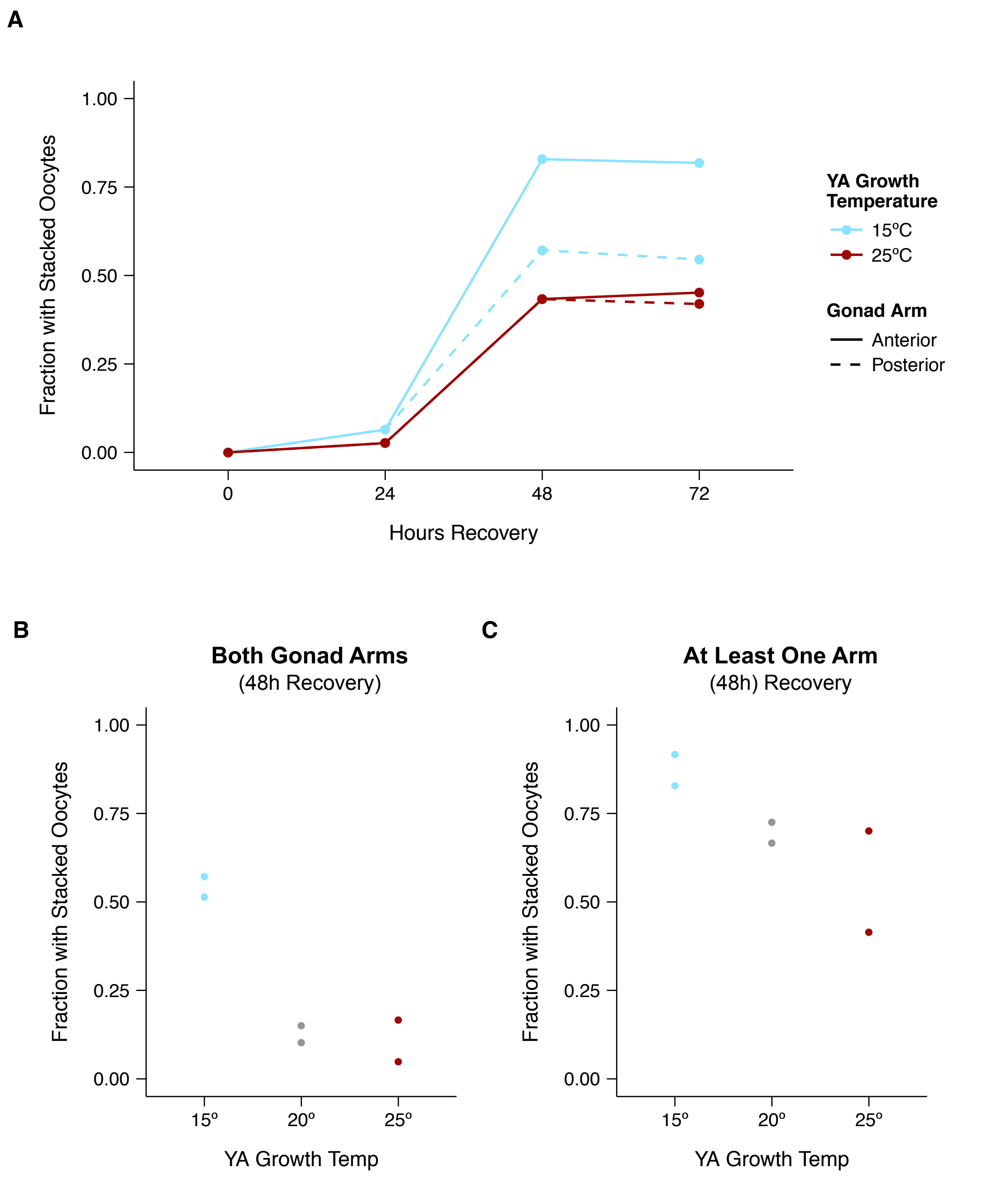

S9 Fig. Gonad damage after 29ºC heat stress. (A) Stacking over time in each gonad arm during recovery from 29ºC heat stress (30 ≤ n ≤ 38 for each time point). (B, C) Individual trials (each with 30 ≤ n ≤ 41) for the stacking results reported in Fig. 6. See S3 Table for raw data.
