# Supplementary material for: Experience Modulates the Reproductive Response to Heat Stress in C. elegans via Multiple Physiological Processes
Source: PLoS One. 2015 Dec 29;10(12):e0145925. doi: 10.1371/journal.pone.0145925 (PMC4699941; doi:10.1371/journal.pone.0145925)
Supplement: S10 Fig — (A) Data from [30] showing the increase in embryonic cell count as a function of time. (B) The same data plotted over a longer interval, with annotations showing important cell movements drawn from [110,114]. See S3 Table for raw data. (PDF) [file pone.0145925.s010.pdf]

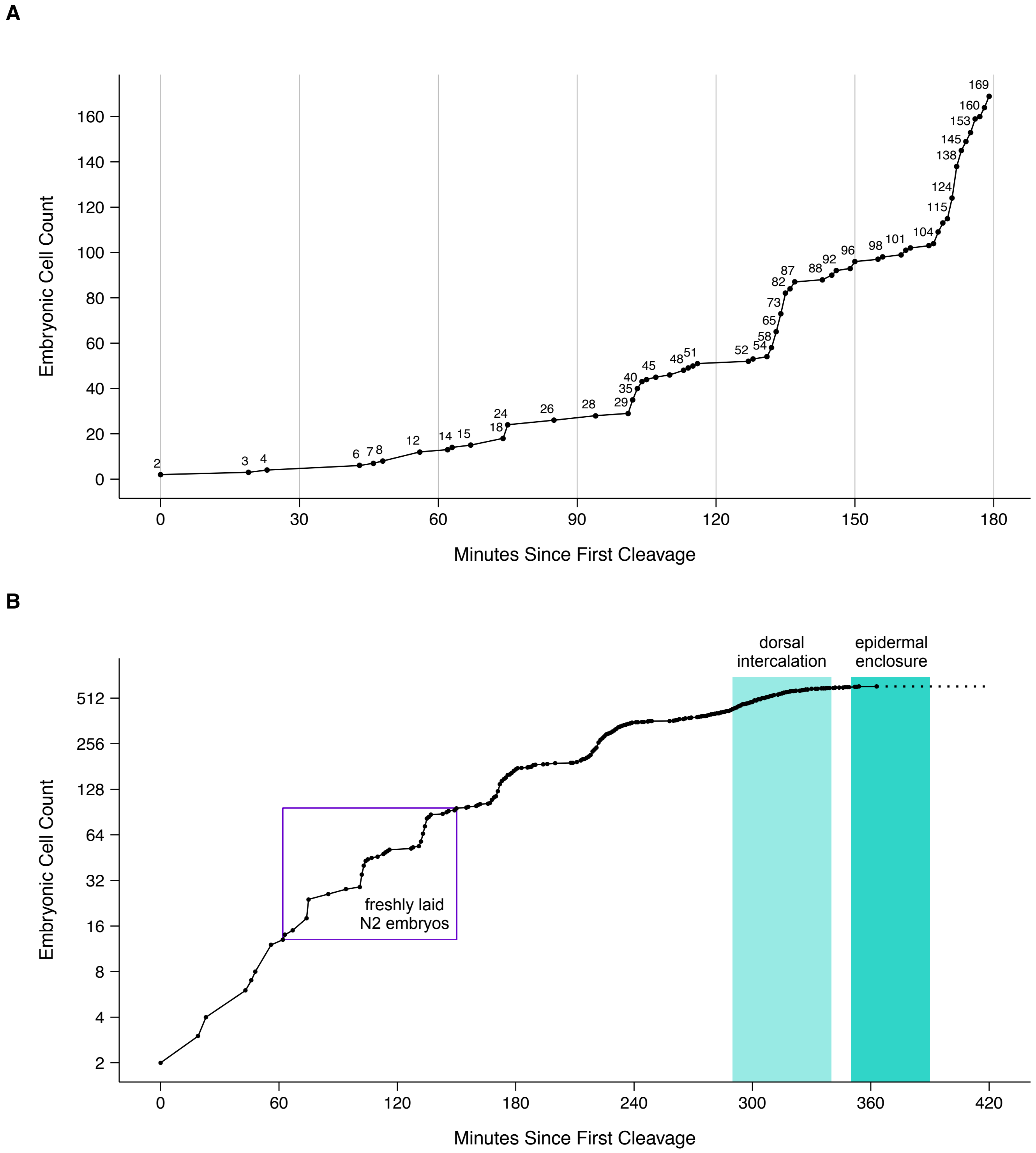

S10 Fig. Embryonic cell counts over time. (A) Data from [30] showing the increase in embryonic cell count as a function of time. (B) The same data plotted over a longer interval, with annotations showing important cell movements drawn from [110,114]. See S3 Table for raw data.
