# Supplementary material for: Experience Modulates the Reproductive Response to Heat Stress in C. elegans via Multiple Physiological Processes
Source: PLoS One. 2015 Dec 29;10(12):e0145925. doi: 10.1371/journal.pone.0145925 (PMC4699941; doi:10.1371/journal.pone.0145925)
Supplement: S11 Fig — (A, B) Individual trials (each with 63 ≤ n ≤ 260) for the hatching data reported in Fig 7B and 7D. Embryos laid during the same collection period are connected with dashed lines. (C) Cell counts of embryos laid by mothers raised at 15°C. (D) A 3-hour delay of heat stress only marginally improved the hatching rate of embryos laid by mothers raised at 15°C. See S3 Table for raw data. (PDF) [file pone.0145925.s011.pdf]

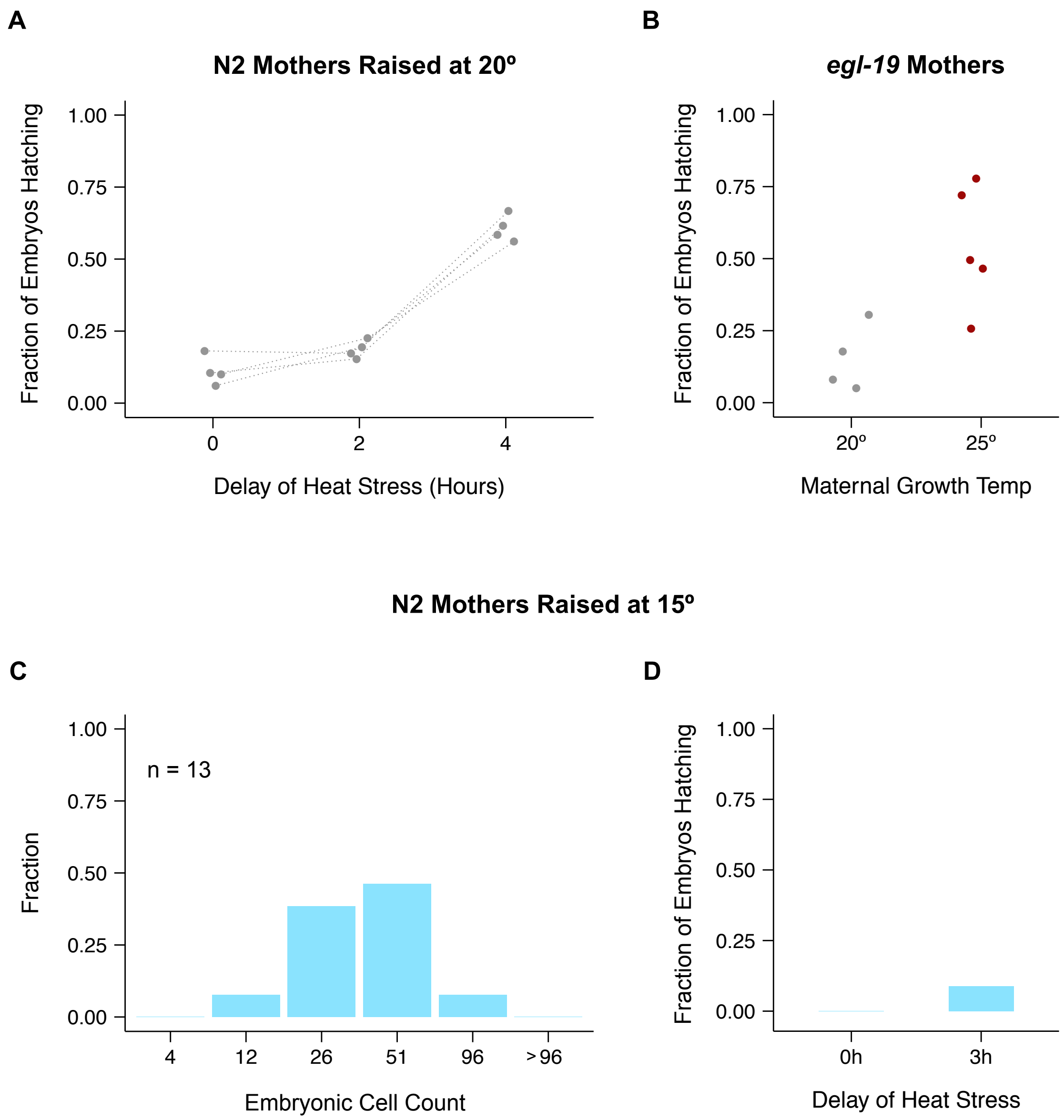

S11 Fig. Effects of maternal cultivation temperature on hatching. (A, B) Individual trials (each with 63 ≤ n ≤ 260) for the hatching data reported in Fig. 7B and 7D. Embryos laid during the same collection period are connected with dashed lines. (C) Cell counts of embryos laid by mothers raised at 15ºC. (D) A 3-hour delay of heat stress only marginally improved the hatching rate of embryos laid by mothers raised at 15ºC. See S3 Table for raw data.
