# Supplementary material for: Experience Modulates the Reproductive Response to Heat Stress in C. elegans via Multiple Physiological Processes
Source: PLoS One. 2015 Dec 29;10(12):e0145925. doi: 10.1371/journal.pone.0145925 (PMC4699941; doi:10.1371/journal.pone.0145925)
Supplement: S12 Fig — (A) Individual experiments (each with n = 50) reported collectively in Fig 8A. (B) Results of a separate experiment in which the F1 offspring of stressed parents were themselves stressed at 29°C (binomial exact test p = 0.044). See S3 Table for raw data. (PDF) [file pone.0145925.s012.pdf]

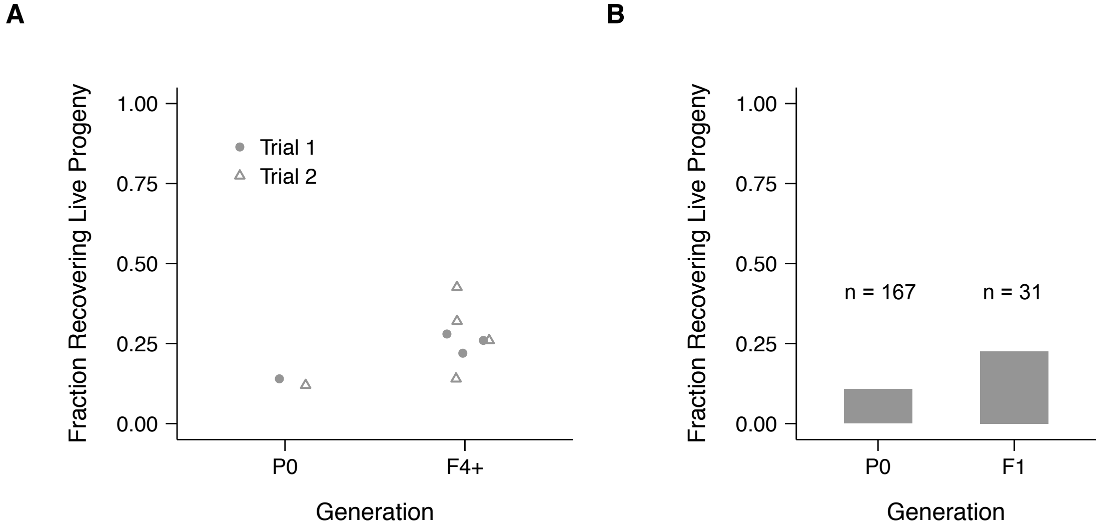

S12 Fig. Hormesis in the descendants of worms raised at 20ºC and stressed at 29ºC.
(A) Individual experiments (each with n = 50) reported collectively in Fig. 8A. (B) Results of a separate experiment in which the F1 offspring of stressed parents were themselves stressed at 29ºC (binomial exact test $p = 0.044$). See S3 Table for raw data.
